# Supplementary material for: Interfacial piezoelectric polarization locking in printable Ti3C2Tx MXene-fluoropolymer composites
Source: Nat Commun. 2021 May 26;12:3171. doi: 10.1038/s41467-021-23341-3 (PMC8155213; doi:10.1038/s41467-021-23341-3)
Supplement: Supplementary file 1 — Supplementary Information [file 41467_2021_23341_MOESM1_ESM.pdf]

Supplementary Information

Interfacial piezoelectric polarization locking in printable $Ti_3C_2T_x$ MXene-fluoropolymer composites

Nick A. Shepelin[1,2,8], Peter C. Sherrell[1,2], Emmanuel N. Skountzos[3,4], Eirini Goudeli[1], Jizhen Zhang[5], Vanessa C. Lussini[6], Beenish Imtiaz[1], Ken Aldren S. Usman[5], Greg W. Dicinoski[6], Joseph G. Shapter[7], Joselito M. Razal[5] & Amanda V. Ellis[1,2]*

[1] Department of Chemical Engineering, The University of Melbourne, Parkville, Victoria 3010, Australia.

[2] BioFab3D, Aikenhead Centre for Medical Discovery, St Vincent's Hospital Melbourne, Fitzroy, Victoria 3065, Australia.

[3] Department of Chemical Engineering, University of Patras, Greece

[4] FORTH/ICE-HT, Patras, GR 26504, Greece.

[5] Institute for Frontier Materials, Deakin University, Geelong, Victoria 3216, Australia.

[6] Note Issue Department, Reserve Bank of Australia, Craigieburn, Victoria 3064, Australia.

[7] Australian Institute for Bioengineering and Nanotechnology, The University of Queensland, Brisbane, Queensland 4072, Australia.

[8] Present address: Laboratory for Multiscale Materials Experiments, Paul Scherrer Institut, Forschungsstrasse 111, CH-5232, Villigen, Switzerland.

* Email: amanda.ellis@unimeb.edu.au

Contents

$Ti_3C_2T_x$ MXene nanosheets and properties 3

Preparation of PVDF-TrFE and $Ti_3C_2T_x$/PVDF-TrFE inks 5

Molecular dynamics (MD) modelling of the interface between the $Ti_3C_2T_x$ nanosheets and the PVDF-TrFE co-polymer 8

Rheological printing optimization of pristine PVDF-TrFE in acetone 16

Steady state rheology 16

Oscillatory rheology 18

Properties of the SEA extrusion printed $Ti_3C_2T_x$/PVDF-TrFE films 23

Mechanical properties 23

Optical properties 25

Raman analysis 27

Attenuated total reflection Fourier transform infrared (ATR-FTIR) spectroscopy 29

X-ray powder diffractometry (XRD) 30

Differential scanning calorimetry (DSC) 33

Piezoresponse force microscopy (PFM) of $Ti_3C_2T_x$/PVDF-TrFE films 35

Macroscale energy harvesting characteristics 42

Piezoelectric generator (PEG) fabrication 42

Macroscale displacement field measurement under compressive stress 44

Measurement of dielectric properties 49

Piezoelectric voltage coefficient and piezoelectric figure of merit 51

References 55

$Ti_3C_2T_x$ MXene nanosheets and properties

The X-ray powder diffraction (XRD) patterns of the Ti_3AlC_2 MAX phase and the $Ti_3C_2T_x$ MXene nanosheets were obtained using a powder diffractometer (X'Pert Powder, PANalytical) equipped with a Cu Kα radiation (40 kV, 30 mA) with an X-ray wavelength (λ) of 1.54 Å at a 2θ scan step of 0.013°. Transmission electron microscopy (TEM) (JEM-2100, JEOL, Ltd.) characterization was employed to study the exfoliated $Ti_3C_2T_x$ nanosheets. Topographical atomic force microscopy (AFM) images were obtained using Bruker's proprietary ScanAsyst scan mode (MultiMode 8-HR, Bruker) to measure the $Ti_3C_2T_x$ nanosheet thickness. AFM samples were prepared by drop casting the $Ti_3C_2T_x$ nanosheet in DMF solutions onto clean silicon wafers. Dynamic light scattering (DLS) was performed using a Zetasizer (Nano ZS, Malvern Instruments) to measure the size distribution of the $Ti_3C_2T_x$ nanosheets. The X-ray photoelectron spectroscopy (XPS) data on the $Ti_3C_2T_x$ nanosheets were acquired using an AXIS Nova (Kratos Analytical Ltd.) equipped with a monochromated Al Kα source (hv = 1486.6 eV) operating at 150 W at a step of 0.1 eV.

The $Ti_3C_2T_x$ MXene nanosheets were synthesized from the Ti_3AlC_2 parent ternary carbide precursor (MAX phase) by selective etching of the aluminium layer (A-group element) using a mixture of lithium fluoride (LiF) and hydrochloric acid (HCl) at room temperature for 24 h.[1–3] The subsequent intercalation of water (H_2O) molecules and Li^+ ions within the negatively charged surface resulted in a volume increase during washing with ultra-pure water, indicating the self-delamination of multi-layered $Ti_3C_2T_x$ to few/single layers.[4] The delamination and the removal of the aluminium was confirmed by the downshifting of the (002) peak and a disappearance of the aluminium peak at 2θ of 39° in the XRD spectra (Supplementary Fig. 1a).[5] The TEM image (Supplementary Fig. 1b) and DLS data (Supplementary Fig. 1c) showed that the $Ti_3C_2T_x$ nanosheets exhibited an average lateral size of approximately 310 nm. The

AFM image showed that the $Ti_3C_2T_x$ nanosheets exhibited a clean surface at the edge (Supplementary Fig. 1d). The thickness profile of the $Ti_3C_2T_x$ nanosheets (Supplementary Fig. 1d, inset) showed an average height of 1.6 nm, corresponding to single-layer $Ti_3C_2T_x$ nanosheets.[1]

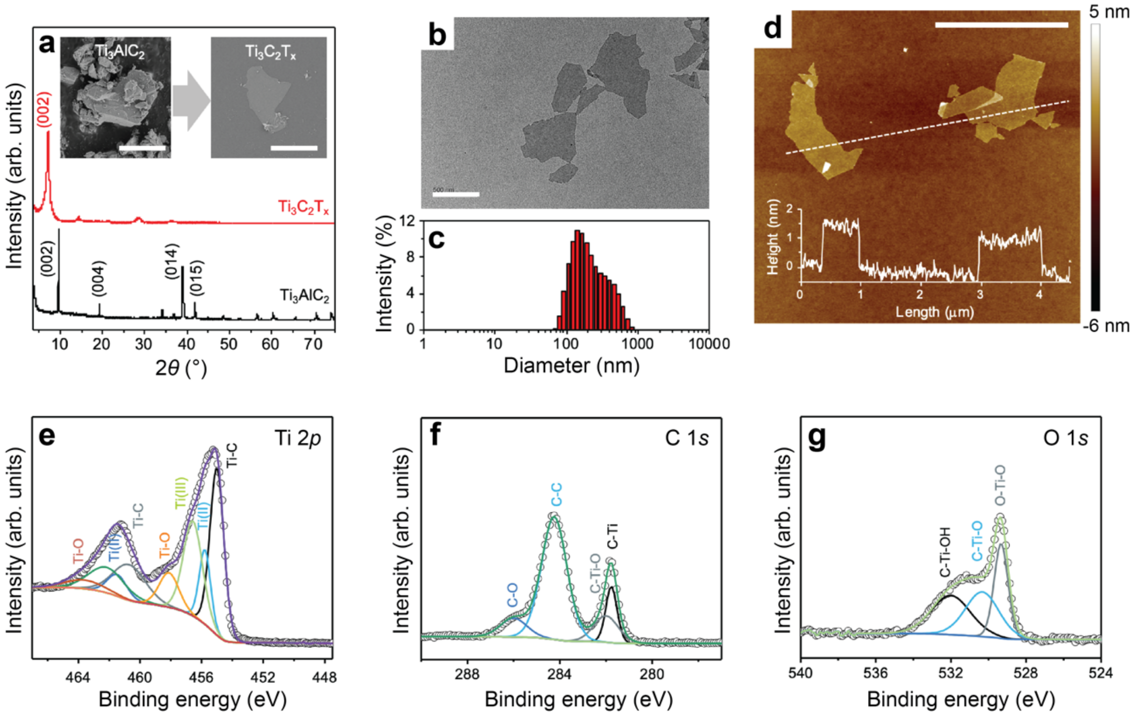

Supplementary Fig. 1: $Ti_3C_2T_x$ MXene nanosheet characterization. **a** XRD pattern and SEM images (insets) of Ti_3AlC_2 MAX phase and $Ti_3C_2T_x$ nanosheets after etching. Scale bar for SEM of Ti_3AlC_2 represents 5 µm and that of $Ti_3C_2T_x$ nanosheets represents 1 µm. **b** TEM image of single-layer 2D $Ti_3C_2T_x$ nanosheets. Scale bar represents 500 nm. **c** The DLS result showing the hydrodynamic size of the $Ti_3C_2T_x$ nanosheets in water. **d** AFM image of the $Ti_3C_2T_x$ nanosheets deposited on a silicon wafer. Inset, represents the thickness profile along the line indicated. Scale bar represents 2 µm. **e-g** Deconvolution of high-resolution XPS spectra for the **e** Ti 2*p*, **f** C 1*s* and **g** O 1*s* orbitals, showing the surface termination and state of the $Ti_3C_2T_x$ nanosheets.

XPS of the $Ti_3C_2T_x$ nanosheets (Supplementary Fig. 1e-g) revealed surface termination dominated by Ti-O and O-Ti-O bonding. The Ti 2*p* region (Supplementary Fig. 1e) showed doublets corresponding to Ti-C, Ti (III), Ti (II) and Ti-O bonding. The dominant Ti-C peak arises from the bridging C atoms between Ti atoms, whereas the Ti-O peaks corresponds to surface termination functional groups of hydroxides (Ti-OH) or epoxide (Ti-O-Ti) structures.[6] The C 1*s* spectral region (Supplementary Fig. 1f), shows four singlet peaks, C-Ti, C-Ti-O, C-

C, and C-O. The C-Ti peak corresponds to internal bridging C atoms. The C-Ti-O peak, occurring at slightly higher binding energies, arises from the long-range influence of oxygen-based surface termination on the electronic state of the internal C atoms. The C-C signal, while anomalous given the crystal structure of $Ti_3C_2T_x$, is always observed in literature[7] and is understood to arise from residual hydrocarbons[8] in the XPS chamber. The C-O peak occurs as Ti is an extremely mobile metal, known to leave vacancies and thus slightly altered stoichiometry.[9] These vacancies result in C-O bonding in the top or bottom Ti metal layer. The O 1*s* region (Supplementary Fig. 1g) confirms the predominant binding of O moieties to Ti atoms in the form of O-Ti-O, which can correspond to either hydroxide (favorable) or epoxide (unfavorable) surface terminations. Higher binding energy peaks for C-Ti-O and C-Ti-OH suggest hydroxy termination is dominant of the surface of the flakes.

Preparation of PVDF-TrFE and $Ti_3C_2T_x$/PVDF-TrFE inks

Recently, we described the dissolution and recycling of SEA extrusion printed PVDF-TrFE co-polymer films using acetone as the only solvent.[10] Here, *N,N*-dimethylformamide (DMF) was completely eliminated as a solvent for extrusion printing entirely and replaced by acetone. Acetone has inherent advantages over DMF and other solvents commonly used to dissolve fluoropolymers, with faster evaporation rates that enable rapid crystallization and drying of SEA extrusion printed polymer films.[11] In particular, DMF exhibits a high boiling point (>150 °C at 101.3 kPa),[12] low vapor pressure (<0.5 kPa at 21 °C)[13] and high toxicity.[12] Comparatively, acetone exhibits a low boiling point (56 °C at 101.3 kPa),[14] high vapor pressure (26 kPa at 21 °C)[13] and reduced toxicity, reported as one of the least toxic industrial solvents,[14] and is thus better suited for SEA extrusion printing.

Initially, pristine PVDF-TrFE inks were prepared in acetone, which were used to optimize the SEA extrusion printing parameters. These inks were prepared by a simple mixing method, whereby PVDF-TrFE powder was slowly added to acetone under mechanical stirring. The pristine PVDF-TrFE co-polymer inks were prepared at PVDF-TrFE co-polymer concentrations of 35 wt%, 40 wt% and 45 wt%, based on the concentrations of inks prepared in the previously reported DMF:acetone solvent mixture.[15] The prepared inks were viscous (Supplementary Fig. 2), moving slower when tilted to a 45° angle as the concentration increased. The rheological optimization of the inks for printing is shown further in this document.

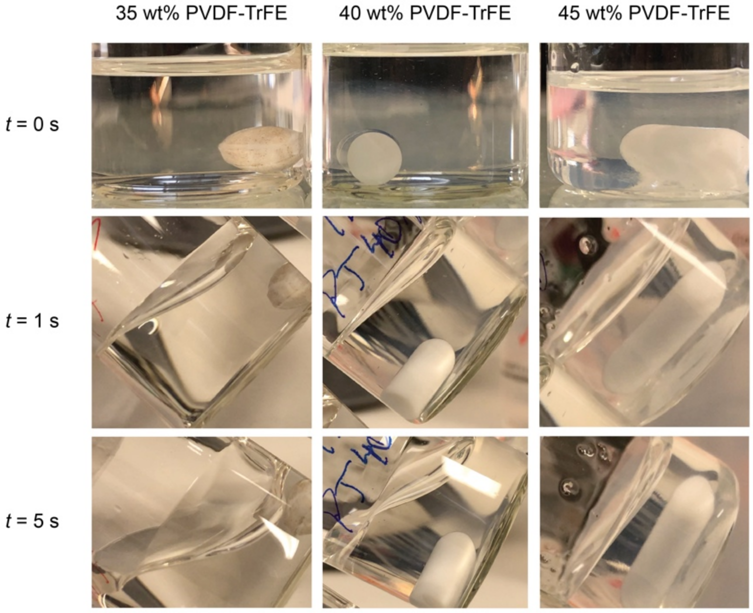

Supplementary Fig. 2: Photographs of the 35 wt% (left column), 40 wt% (middle column) and 45 wt% (right column) PVDF-TrFE/acetone inks, demonstrating the viscosity of the inks before rotating the vial to 45° (top row, *t* = 0 s), 1 s after rotating the vial (middle row) and 5 s after rotating the vial (bottom row).

Similarly, $Ti_3C_2T_x$/PVDF-TrFE inks were prepared at $Ti_3C_2T_x$ concentrations at 0.02 wt%, 0.10 wt%, 0.20 wt% and 0.50 wt%. Here, a small aliquot of the $Ti_3C_2T_x$ stock dispersion in DMF (4.4 mg mL^{-1}) was added to acetone to form dispersions at 0.00 mg mL^{-1}, 0.10 mg mL^{-1}, 0.52 mg mL^{-1}, 1.05 mg mL^{-1} and 2.61 mg mL^{-1} in acetone. Subsequently, the PVDF-TrFE powder was added slowly to the $Ti_3C_2T_x$ dispersions in acetone at 23 °C while stirring, at 40 wt% relative to the mass of the dispersion, to form the $Ti_3C_2T_x$/PVDF-TrFE inks. The inks were

stirred until homogeneous, then sealed with parafilm and stored at -5 °C to minimize solvent evaporation.

Throughout the experimental procedure, the stability of the $Ti_3C_2T_x$ nanosheet dispersion was monitored in the $Ti_3C_2T_x$/PVDF-TrFE ink, for up to five months (Supplementary Fig. 3, middle). The $Ti_3C_2T_x$/PVDF-TrFE ink was compared to a single-walled carbon nanotube (SWCNT)/PVDF-TrFE ink, which we have recently reported (Supplementary Fig. 3, right).[10] Notably, after five months of storage, all three inks exhibited similar flow properties to recently prepared inks. The SWCNTs were found to aggregate in the SWCNT/PVDF-TrFE ink, causing occasional blocking of the nozzle during printing. Conversely, the $Ti_3C_2T_x$ nanosheets showed minimal aggregation in the $Ti_3C_2T_x$/PVDF-TrFE ink due to exceptional electrostatic interactions between the $Ti_3C_2T_x$ nanosheets and the PVDF-TrFE co-polymer (Supplementary Fig. 5, Supplementary Movie 1). The $Ti_3C_2T_x$/PVDF-TrFE ink could be printed following long-term storage with no required changes in the extrusion printing parameters.

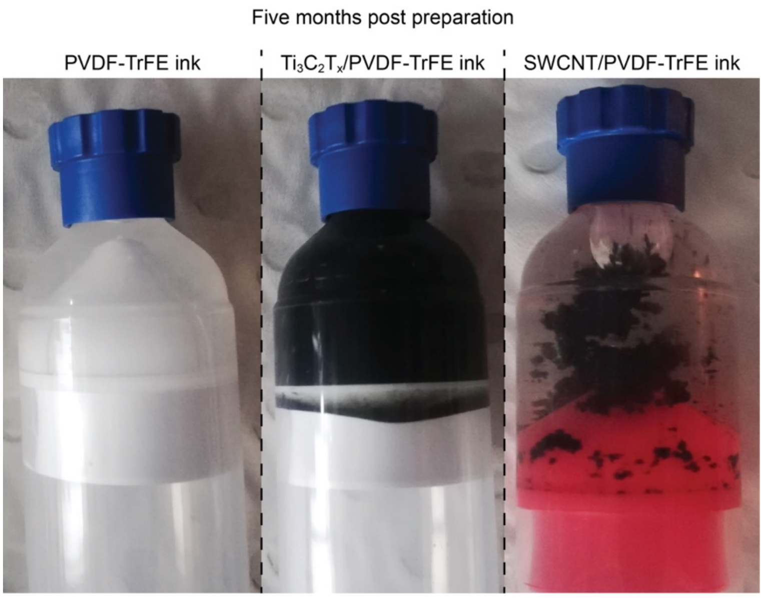

Supplementary Fig. 3: Comparison of the stability of the PVDF-TrFE ink (left), $Ti_3C_2T_x$/PVDF-TrFE ink (middle) and SWCNT/PVDF-TrFE ink (right), five months after preparation of the ink.

Molecular dynamics (MD) modelling of the interface between the $Ti_3C_2T_x$ nanosheets and the PVDF-TrFE co-polymer

The density of the PVDF-TrFE co-polymer melt was investigated as a function of the monomer units (alternatively the molecular weight) to validate the interatomic potential used for the simulations (Supplementary Fig. 4). The density was found to increase with increasing number of monomer units, reaching an asymptotic plateau corresponding to 1.42 g cm^{-3}. The value obtained using MD simulations was in excellent agreement with the value of 1.49 g cm^{-3} provided by the manufacturer of the PVDF-TrFE co-polymer (Solvay), validating the interatomic potential used in the MD simulations.

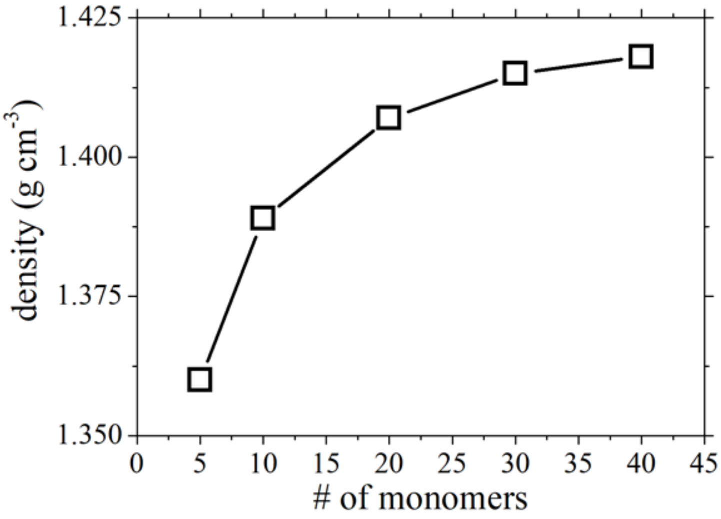

Supplementary Fig. 4: Evolution of the density of the PVDF-TrFE co-polymer melt as a function of the number of monomers.

The distribution of the local density of the PVDF-TrFE co-polymer film (70 chains) was investigated as a function of distance from the substrate, for a graphene substrate (Supplementary Fig. 5, black line) and a $Ti_3C_2T_x$ nanosheet substrate (Supplementary Fig. 5, red line). The local density distribution was calculated from the mass within each separation distance interval, normalized to the volume within said interval and averaged over the duration

of the simulation (1.8 ns timespan). The shaded regions correspond to the minimum and maximum values of the local density at each separation distance interval relative to the graphene or $Ti_3C_2T_x$ nanosheet substrate. The layer adjacent to the substrate was found to adsorb to both the graphene and the $Ti_3C_2T_x$ nanosheet substrates, exhibiting a local density of approximately 2.3 g cm^{-3} and 1.6 g cm^{-3}, respectively. The larger local density of the PVDF-TrFE co-polymer film adjacent to the graphene substrate indicates that the PVDF-TrFE co-polymer chains are more packed than those adjacent to the $Ti_3C_2T_x$ nanosheet substrate, as the latter possesses an increased surface roughness due to the OH termination, thus inducing steric effects in the PVDF-TrFE co-polymer chains. The local density of the layers further away from the substrate is practically identical in both the $Ti_3C_2T_x$/PVDF-TrFE and the graphene/PVDF-TrFE systems. Notably, the PVDF-TrFE co-polymer film adsorbs closer to the $Ti_3C_2T_x$ nanosheet relative to graphene, as the first local density peak appears at a lower separation. This decreased separation indicates a stronger attractive interaction between the $Ti_3C_2T_x$ nanosheet and the PVDF-TrFE co-polymer in comparison to the graphene substrate.

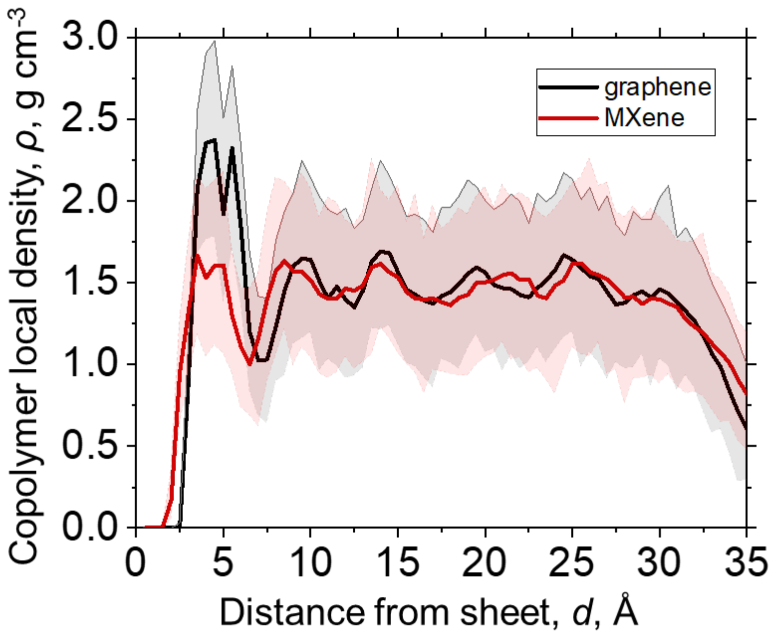

Supplementary Fig. 5: Distribution of the local density of the PVDF-TrFE co-polymer film (70 chains) as a function of the distance from the $Ti_3C_2T_x$ nanosheet (red line) and the graphene sheet (black line) substrates. The overlay represents the minimum and maximum values throughout the simulation.

This enhanced adhesion phenomenon was investigated by adhesion strength studies. Here, a PVDF-TrFE co-polymer chain was placed in close proximity to either the $Ti_3C_2T_x$ nanosheet or graphene sheet substrate. A force was applied to the PVDF-TrFE co-polymer perpendicular to the basal plane of the substrate to measure the desorption force. The force was increased from 0.00 pN to 6.95 pN with a step size of 0.695 pN, monitoring the position of the PVDF-TrFE co-polymer chain for the desorption from the substrate. It was found that the PVDF-TrFE co-polymer chain desorbed form the graphene substrate at approximately 2.78 pN, whereas the required desorption force increased on the $Ti_3C_2T_x$ nanosheet to approximately 4.17 pN, indicating a greater adhesion strength at the interface between the $Ti_3C_2T_x$ nanosheet and the PVDF-TrFE co-polymer.

The distribution of the H and F atoms in the PVDF-TrFE co-polymer film was further investigated as a function of the separation from the $Ti_3C_2T_x$ nanosheet substrate to investigate whether preferential orientation of these dipolar atoms in the PVDF-TrFE co-polymer were giving rise to the polarization locking mechanism (Supplementary Fig. 6). The datapoints represent average values for 14 PVDF-TrFE co-polymer chains and the shaded areas represent the minimum and maximum number of H and F atoms over the entire simulation. The H and F probability distributions were observed to be approximately equal at all separations from the $Ti_3C_2T_x$ nanosheet substrate, indicating the PVDF-TrFE did not preferentially orient on the substrate. Small deviations at a low separation (up to 2 Å) were observed, whereby the H atoms were found closer to the $Ti_3C_2T_x$ nanosheet substrate relative to the F atoms. This was attributed to the shortest non-covalent hydrogen bonds between the H atoms of the PVDF-TrFE co-polymer and the hydroxyl terminations (T_x) of the $Ti_3C_2T_x$ nanosheet substrate.

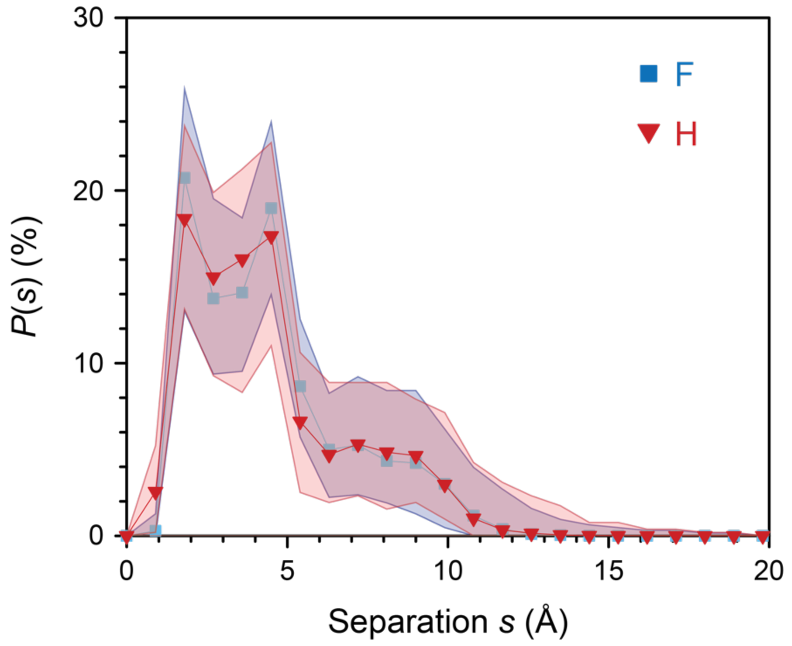

Supplementary Fig. 6: Probability distribution ($P(s)$) of the number of F (squares) and H atoms (triangles) within the PVDF-TrFE co-polymer film (14 chains) as a function of the distance from the $Ti_3C_2T_x$ nanosheet substrate (s). The shaded areas represent the minimum and maximum values over the entire simulation.

To understand the phase distributions of the PVDF-TrFE co-polymer film adjacent to the $Ti_3C_2T_x$ nanosheet substrate, the probability distributions of the dihedral angles were monitored for a 14-chain PVDF-TrFE co-polymer film (Supplementary Fig. 7). The PVDF-TrFE co-polymer consists of three commonly found phases, namely the α phase (non-polar), γ phase (semi-polar) and the β phase (highly polar).[16] These phases correspond to spatial conformation of the bonds, either *trans* (T) or *gauche* (G). The α phase is thermodynamically favored in fluoropolymers, due to its *trans-gauche* (TGTG'TGTG') conformation, which consists of 50% *trans* bonds and 50% *gauche* bonds.[17] Conversely, the β phase is an all-*trans* (TTTTTTTT) conformation, which spatially separates the H moieties on one C atom from the F atoms on the adjacent C atom, giving rise to a strong H-F dipole moment.[18] The γ phase is a stable intermediate state between the α phase and the β phase, as evidenced by the 75% *trans* and 25% *gauche* fraction (TTTGTTTG'), giving rise to dipole moments which result in a lower maximum polarization relative to the β phase.[17] Hence, the distribution of the dihedral angles

and subsequently the phase fractions can provide insight on the changes in local electroactivity of the PVDF-TrFE co-polymer film adjacent to the $Ti_3C_2T_x$ nanosheet substrate.

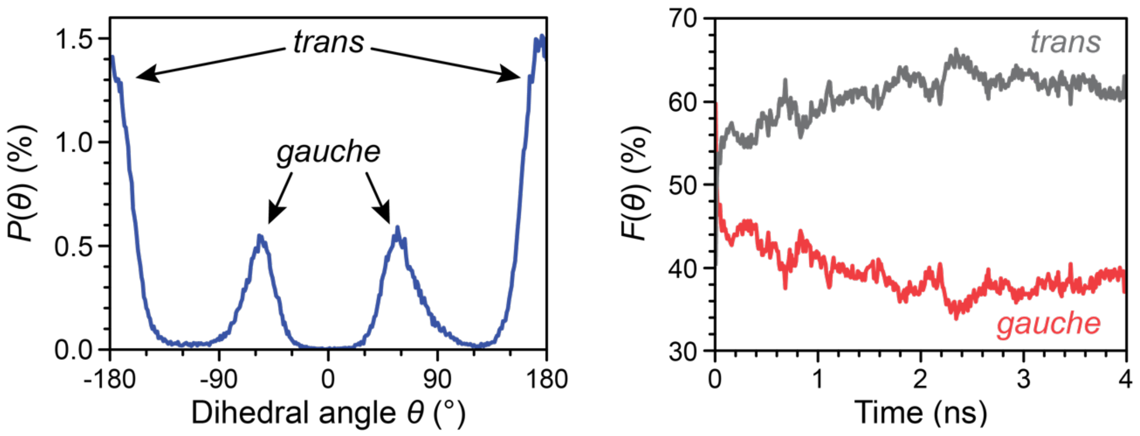

Supplementary Fig. 7: Bond angle distributions of the PVDF-TrFE co-polymer on the $Ti_3C_2T_x$ nanosheet substrate. **a** Probability distribution ($P(\theta)$) of the dihedral angles averaged oven 4 ns for a film of 14 PVDF-TrFE chains interacting with an immobile $Ti_3C_2T_x$ nanosheet. **b** Time evolution of the fraction ($F(\theta)$) of the *trans* (gray line) and *gauche* angles (red line).

The distribution of the dihedral angles (Supplementary Fig. 7a) was taken as the average of all angles in a 14-chain PVDF-TrFE co-polymer film interacting with an immobile $Ti_3C_2T_x$ nanosheet substrate over the span of the simulation (4 ns). The PVDF-TrFE co-polymer chains exhibited configurations at four main dihedral angles, ±180° (*trans*) and ±60° (*gauche*).[18] As a function of simulation time, the fraction of *trans* conformation (Supplementary Fig. 7b, black line) was found to increase and attain a final value of approximately 63%, whereas approximately 37% of the bonds were observed in the *gauche* conformation (Supplementary Fig. 7, red line). These values correspond to either a majority of α phase (74%) with low prevalence of the β phase (26%), or a near-even distribution of the α phase (48%) and gamma phase (52%), or a combination of the two. Importantly, while the PVDF-TrFE generally crystallizes into the β phase due to the third F atom in the TrFE monomer, these values suggest a large presence of the α phase.[10] Indeed, at the local level, the experimental data observed the presence of the α and γ phases adjacent to the $Ti_3C_2T_x$ nanosheet (Fig. 4d, e); however, the

FTIR (Supplementary Fig. 16) and XRD (Supplementary Fig. 17, Supplementary Fig. 18) data presented further in this document suggest the β phase as the primary conformation in the bulk of the $Ti_3C_2T_x$/PVDF-TrFE composites.

Similarly, the temporal evolution of the dihedral angles was repeated for a 70-chain PVDF-TrFE co-polymer film on the $Ti_3C_2T_x$ nanosheet or graphene substrate (Supplementary Fig. 8). Similar to the 14-chain PVDF-TrFE co-polymer films (Supplementary Fig. 7b), the larger films on a $Ti_3C_2T_x$ nanosheet substrate exhibited a larger *trans* fraction (approximately 65%) relative to the *gauche* fraction (approximately 35%). Interestingly, when simulated adjacent to a graphene substrate, the same 70-chain PVDF-TrFE copolymer film exhibited a lower fraction of *trans* bonds (approximately 57%) and subsequently a higher fraction of *gauche* bonds (approximately 43%).

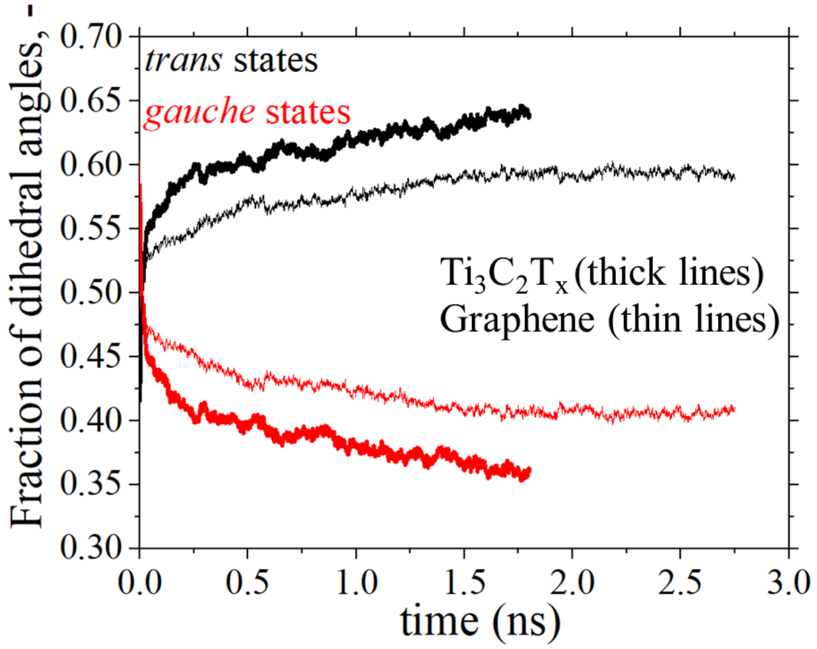

Supplementary Fig. 8: The temporal evolution of the fraction of *trans* bond angles (black lines) and *gauche* bond angles (red lines) in the 70-chain PVDF-TrFE co-polymer film on a $Ti_3C_2T_x$ nanosheet substrate (thick lines) and graphene substrate (thin lines).

The evolution of the polarization angle (θ) in the PVDF-TrFE co-polymer film relative to the basal plane of the $Ti_3C_2T_x$ nanosheet substrate was investigated as a function of the simulation time and the distance from the substrate (Supplementary Fig. 9). The θ was obtained as a function of time for PVDF-TrFE co-polymer chains within 24 Å (green line), 29 Å (red line), 34 Å (blue line) and 39 Å (grey line) from the $Ti_3C_2T_x$ nanosheet substrate. The PVDF-TrFE co-polymer chains closer to the substrate (<24 Å) exhibited a broad range of θ values, found to sporadically change orientation throughout the simulation. Conversely, as the separation from the $Ti_3C_2T_x$ increased to 39 Å, the θ was found to orient perpendicular to the basal plane of the $Ti_3C_2T_x$. Additionally, the orientation of the polarization vector at 39 Å was not found to significantly deviate from the perpendicular orientation throughout the equilibrated region (t > 0.5 ns) of the simulation, indicating that the polarization locking spontaneously occurs near the interface of the two materials directly upon contact (in solution) and maintains the perpendicular orientation in the solid state (after deposition).

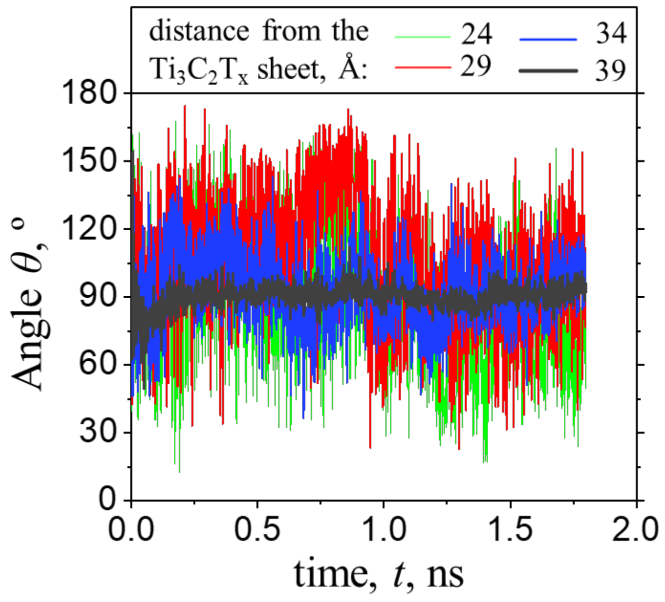

Supplementary Fig. 9: Time evolution of the angle (θ) between the total dipole vector (polarization) in the PVDF-TrFE co-polymer film and the $Ti_3C_2T_x$ nanosheet basal plane, for PVDF-TrFE co-polymer chains that are within 24 Å (green line), 29 Å (blue line), 34 Å (blue line) and 39 Å (grey line) from the $Ti_3C_2T_x$ nanosheet substrate.

The polarization vectors of five individual PVDF-TrFE co-polymer chains are shown exemplarily for the $Ti_3C_2T_x$ and graphene substrates in Supplementary Movie 2, indicating a correlation between their structure and their corresponding polarization vector. Here, the rest of the PVDF-TrFE co-polymer chains (65 chains out of the 70mer) are omitted for clarity. The chains on the $Ti_3C_2T_x$ substrate are clearly more elongated than those on the graphene, attaining individual chain polarization vectors perpendicular to the basal plane $Ti_3C_2T_x$, corresponding well to the total polarization vector of the 70-chain film. Contrary to the PVDF-TrFE co-polymer chains on the $Ti_3C_2T_x$ nanosheet substrate, those on top of the graphene substrate exhibit coiled morphology with the individual polarization vectors attaining randomized orientations.

Rheological printing optimization of pristine PVDF-TrFE in acetone

To optimize the ink system (PVDF-TrFE in acetone) for SEA 3D printing, the rheological properties of the inks were first studied for PDVF-TrFE (35 wt%, 40 wt% and 45 wt%) loadings. PVDF-TrFE powder (75 mol% VDF, 25 mol% TrFE, M_w = 420 kDa) was slowly added into acetone and stirred at 23 °C until the powder completely dissolved, forming viscous inks (Supplementary Fig. 2). The rheology of these inks was assessed using an MCR 702 rheometer (Anton Paar GmbH) in a cone-plate geometry, with a cone diameter of 25 mm, a cone angle of 2° and a gap at 102 μm (CP25-2, Anton Paar GmbH). The temperature in all measurements was held at 5°C.

Steady state rheology

Initial steady-state logarithmic shear rate ramps were used to compare the viscosity (η) of the PVDF-TrFE ink in acetone to that of the commonly reported solvent mixture of DMF and acetone (40:60 vol%), with polymer concentration at 35 wt% (Supplementary Fig. 10a).[10,15] Both inks showed non-Newtonian (shear thinning) behavior, which is required for extrusion printing.[19] At a shear rate of 0.01 s^{-1}, which corresponded to the resting state (prior to and post printing), the η of the ink in the DMF:acetone solvent system was measured at 670 Pa s, drastically lower than that of the ink in acetone as the solvent, measured at 430,000 Pa s. The extreme increase in the viscosity at low shear represents a three order of magnitude increase in shape retention capability of the ink directly upon printing, further aided by the faster evaporation rate of acetone relative to DMF. Interestingly, the viscosity of the ink with acetone as the only solvent exhibited a lower η (7 Pa s) at high shear rate (1,000 s^{-1}, corresponding to conditions during printing) relative to the ink with DMF:acetone as the solvent system (12 Pa

s). This signifies a lower pressure is required to extrude the same volume of ink, following the Hagen-Poiseuille equation.[20] This initial testing confirmed the significant improvement in the rheological properties of the PVDF-TrFE/acetone ink relative to the PVDF-TrFE/(DMF:acetone) ink and further suggested the formation of a gel, consistent with prior reports of acetone as a swelling agent for fluoropolymers.[21]

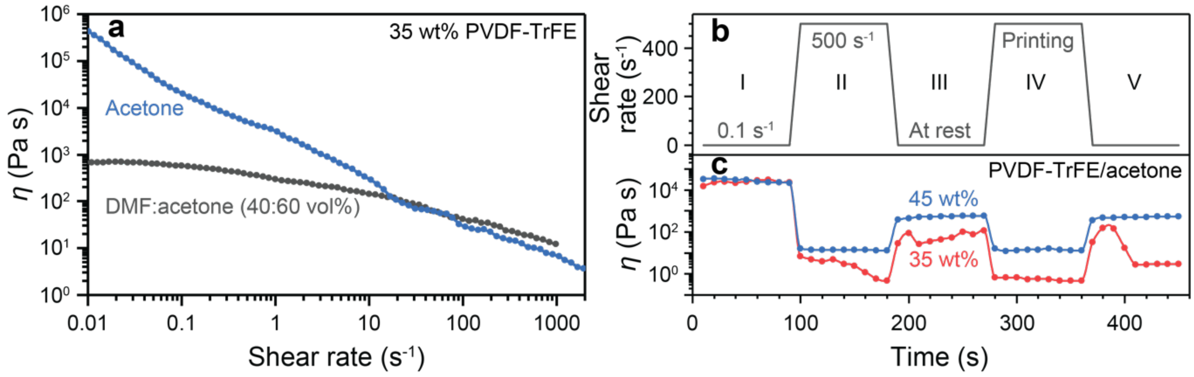

Supplementary Fig. 10: Steady-state rheology. **a** The shear rate sweeps for inks of PVDF-TrFE/(DMF:acetone, 40:60 vol%) and PVDF-TrFE/acetone, containing PVDF-TrFE (35 wt%). **b** The applied shear rate profile as a function of time and **c** the resultant viscosity for PVDF-TrFE/acetone inks with PVDF-TrFE concentration at 35 wt% and 45 wt%.

Additional steady state testing was undertaken on the PVDF-TrFE/acetone ink at two concentrations, 35 wt% and 45 wt%, to simulate the flow and recovery parameters found during extrusion printing (Supplementary Fig. 10b, c). Here, the shear rate was kept constant for 90 s, cycling between 1 s^{-1} and 500 s^{-1} (Supplementary Fig. 10b), and measuring the η (Supplementary Fig. 10c). Both inks exhibited similar η values in region I (between 21,000 Pa s and 23,000 Pa s at 90 s), decreasing under increased shear in region II to 16 Pa s in the 45 wt% ink and 7 Pa s in the 35 wt% ink. Notably, the η of the 35 wt% ink exhibited further decreases at the constant shear rate to 0.5 Pa s, which is likely to arise from elongation and disentanglement of the polymer chains. Region III showed partial recovery in both inks to 588 Pa s (2.7%) and 113 Pa s (0.5%) in the 45 wt% and 35 wt% inks, respectively. This suggests the higher concentration of PVDF-TrFE assists in stabilizing the entanglement in the polymer

chains; however, the application of shear nonetheless reduces the entanglement between the polymer chains, correlating to a pseudo-1D material.[22] In region IV, the 35 wt% ink was found to drop in η to the lower value of that in region II, suggesting that the disentanglement is irreversible, whereas the η of the 45 wt% ink was found to be consistent throughout the region, with the same values as region II. Surprisingly, upon decrease in shear rate in region V, the η of the 35 wt% ink decreased to similar values as the high shear rate region II, unable to reliably recover to the values of region III, confirming the disentanglement effects and therefore proving unsuitable for a printing system where the printed ink must retain its shape.

Oscillatory rheology

Oscillatory rheology was employed at 5 °C to further probe the hypothesis of gel formation and determine the flow parameters in the PVDF-TrFE/acetone inks with PVDF-TrFE concentration at 35 wt%, 40 wt% and 45 wt% (Supplementary Fig. 11).[19] Oscillatory frequency (ω) sweeps were performed (Supplementary Fig. 11a-c), which can give insight into the time-dependent flow properties of the inks.[23] The tests were performed with fixed shear strain (γ_s) at 1%. All measured samples exhibited a similar trend in the storage (G') and loss (G'') moduli as a function of the ω, confirming the increased η (Supplementary Fig. 10a) relative to PVDF-TrFE/(DMF:acetone) inks was due to enhanced swelling of the fluoropolymer, which has lower dependence on the fluoropolymer concentration.[24] Furthermore, minimal deviation in the slope of G' and G'' over the entire measured ω range strongly suggested the formation of a strongly bound gel, which was solid-like ($G' > G''$) for all measured frequencies.[23]

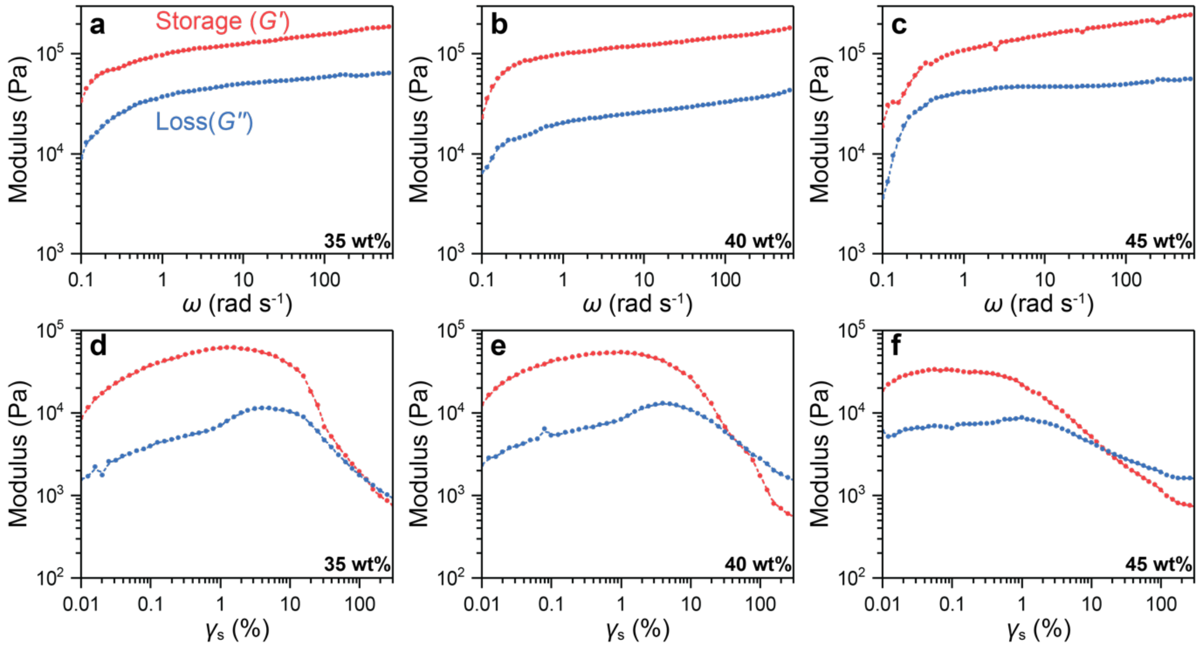

Supplementary Fig. 11: Oscillatory rheology measurements on PVDF-TrFE/acetone inks. **a-c** The frequency (ω) sweeps for **a** 35 wt%, **b** 40 wt% and **c** 45 wt% PVDF-TrFE/acetone inks, obtained at fixed strain (γ_s = 1%). **d-f** The γ_s sweeps for **d** 35 wt%, **e** 40 wt% and **f** 45 wt% PVDF-TrFE/acetone inks, obtained at fixed frequency (ω = 1 Hz).

Similarly, the oscillatory γ_s sweeps (Supplementary Fig. 11d-f) at low ω (1 Hz) exhibited similar characteristics between all three PVDF-TrFE concentrations in acetone. All three tested samples showed solid-like behavior ($G' > G''$) at low γ_s, followed by a liquid-like region ($G' < G''$) at high γ_s (>100%).[19] Interestingly, the flow point (γ_s at cross-over of G' and G'') was found to decrease with increasing concentration, which, while counterintuitive, suggests the strong binding between the PVDF-TrFE and acetone.[25] As the concentration increases, the number of polymer-solvent contact points decreases (increasing polymer-polymer binding points), therefore the gel becomes weakened and is able to flow with a lower γ_s. In translating this theory to extrusion printing, all of the three tested inks were suitable for printing; however, a lower flow point would decrease the required pressure input to extrude the sample, meaning the inks with higher PVDF-TrFE concentration are preferred for the printing.[20]

Finally, oscillatory shear stress (σ_s) cycling was undertaken to probe the recovery parameters of G' and G'' within the inks and determine the optimal PVDF-TrFE concentration (35 wt%, 40 wt% or 45 wt%) in acetone for extrusion printing (Supplementary Fig. 12).[19] Here, the σ_s was cycled at constant ω (1 Hz) between 1 Pa and 5 kPa, representing the induced σ_s at rest and during printing, respectively, held constant for at least 70 s (Supplementary Fig. 12a). At PVDF-TrFE (35 wt%), the ink was unable to maintain σ_s at 6 kPa, whereas the inks containing 40 wt% and 45 wt% PVDF-TrFE exhibited consistent response to the input σ_s. The value for the tan(δ), or the ratio of G'' and G' was <1 (marked by grey horizontal line) for all samples at 1 Pa σ_s and increased to > 1 upon the application of 5 kPa σ_s for 70 s (Supplementary Fig. 12b). For the inks containing 40 wt% and 45 wt% PVDF-TrFE, the tan(δ) remained constant throughout the 5 kPa σ_s region and completely recovered for all concentrations after 70 s at 1 Pa σ_s. During the second cycle, the 35 wt% ink was found to flow with the lowest resistance, represented by a tan(δ) value of 20,000 (instrument limit), whereas the 40 wt% and 45 wt% inks retained similar values to the first cycle. Supplementary Fig. 12c shows the complex viscosity (η^*) of the PVDF-TrFE inks. As expected, the starting η^* was found to increase with increasing PVDF-TrFE concentration. During the first high σ_s cycle, the η^* was found to decrease significantly for the 35 wt% ink as a function of time and unable to recover to initial values in the subsequent low stress cycle. Conversely, the 40 wt% and 45 wt% PVDF-TrFE inks exhibited full recovery after two high stress cycles.

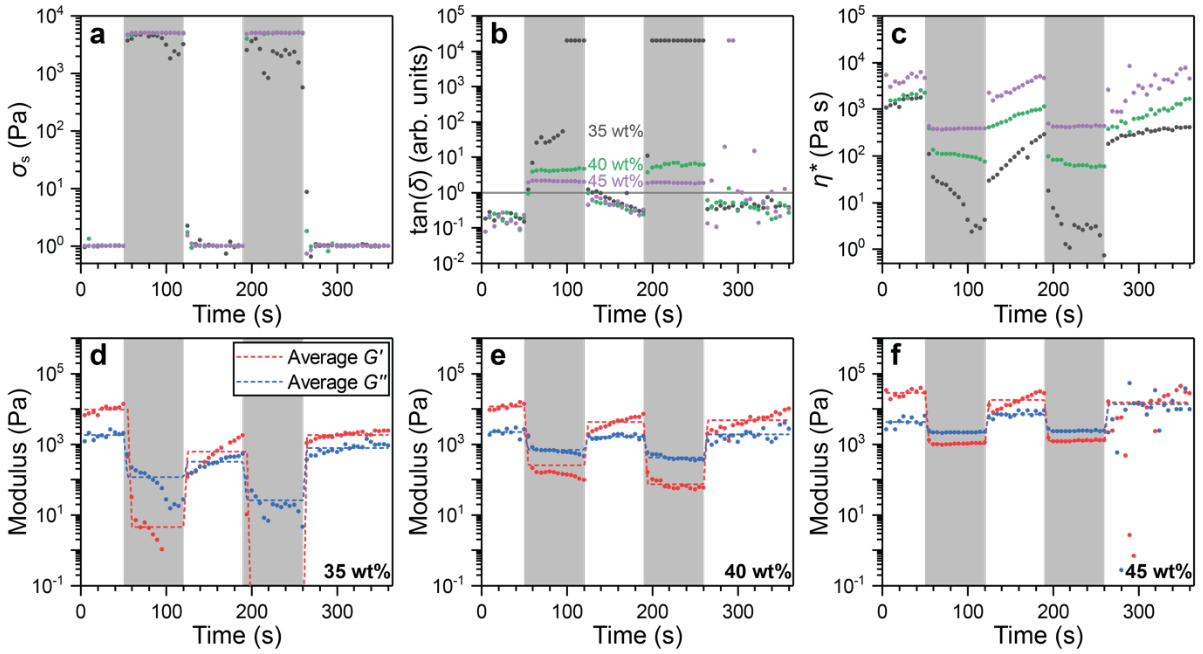

Supplementary Fig. 12: Time-dependent oscillatory shear stress cycling for the PVDF-TrFE/acetone (35 wt%, 40 wt% and 45 wt%) inks. **a** The applied oscillatory shear stress (σ_s) with the high stress regions shaded grey. **b** The tan(δ) response to the shear stress with the liquid-solid transition point (tan(δ) = 1) represented by a solid grey line. **c** The complex viscosity (η*) response to the shear stress. **d-f** The storage (G') and loss (G'') moduli response to the shear stress for **d** 35 wt% PVDF-TrFE/acetone ink, **e** 40 wt% PVDF-TrFE/acetone ink and **f** 45 wt% PVDF-TrFE ink, performed with ω at 1 Hz.

The temporal evolution of the G' and G'' during the σ_s cycling is shown in Supplementary Fig. 12d-f for the three concentrations of PVDF-TrFE in acetone. For the 35 wt% ink (Supplementary Fig. 12d), the G' was observed to decrease rapidly as a function of time at σ_s = 5 kPa, with an average decrease over the timespan of greater than 1,000-fold. In the subsequent low-σ_s period, the slope of G' was higher than that of G''; however, the G' was unable to recover to the initial value of 9,600 Pa, reaching a maximum of 1,736 Pa. Throughout the second σ_s = 5 kPa cycle, the G' for the 35 wt% ink decreased significantly to below 1 mPa and subsequently exhibited a significantly lower slope during recovery. Conversely, the 40 wt% (Supplementary Fig. S12e) and 45 wt% (Supplementary Fig. 12f) PVDF-TrFE inks were stable under high-σ_s for at least one cycle and showed considerably higher G' recovery relative to the PVDF-TrFE (35 wt%) ink, from initial values of 11,800 Pa and 28,300 Pa, to final maxima of 7,000 Pa and 27,500 Pa, respectively. While the PVDF-TrFE (40 wt%) ink

exhibited similar characteristics throughout the second σ_s = 5 kPa cycle (Supplementary Fig. 12e), the PVDF-TrFE (45 wt%) ink was unable to consistently recover to initial values (Supplementary Fig. 12f). Therefore, PVDF-TrFE (40 wt%) ink was selected for further experiments involving the incorporation of $Ti_3C_2T_x$ nanosheets.

Properties of the SEA extrusion printed $Ti_3C_2T_x$/PVDF-TrFE films

Mechanical properties

The tensile mechanical properties of the SEA extrusion printed $Ti_3C_2T_x$/PVDF-TrFE films were measured by a dynamic mechanical tester (ElectroForce 5500, TA Instruments). Samples, with a length of 27 mm and a width of 5 mm (Supplementary Fig. 13a), were secured in grips by friction adhesive, with the distance between grips set at 5 mm (Supplementary Fig. 13b). The width (*w*) and thickness (*t*) of each sample is given in Supplementary Table 1. The films were extended parallel to the printing axis at a rate of 0.01 mm s^{-1}. Notably, the instrument displacement limit was approximately 11 mm, significantly below the breaking strain of the sample.

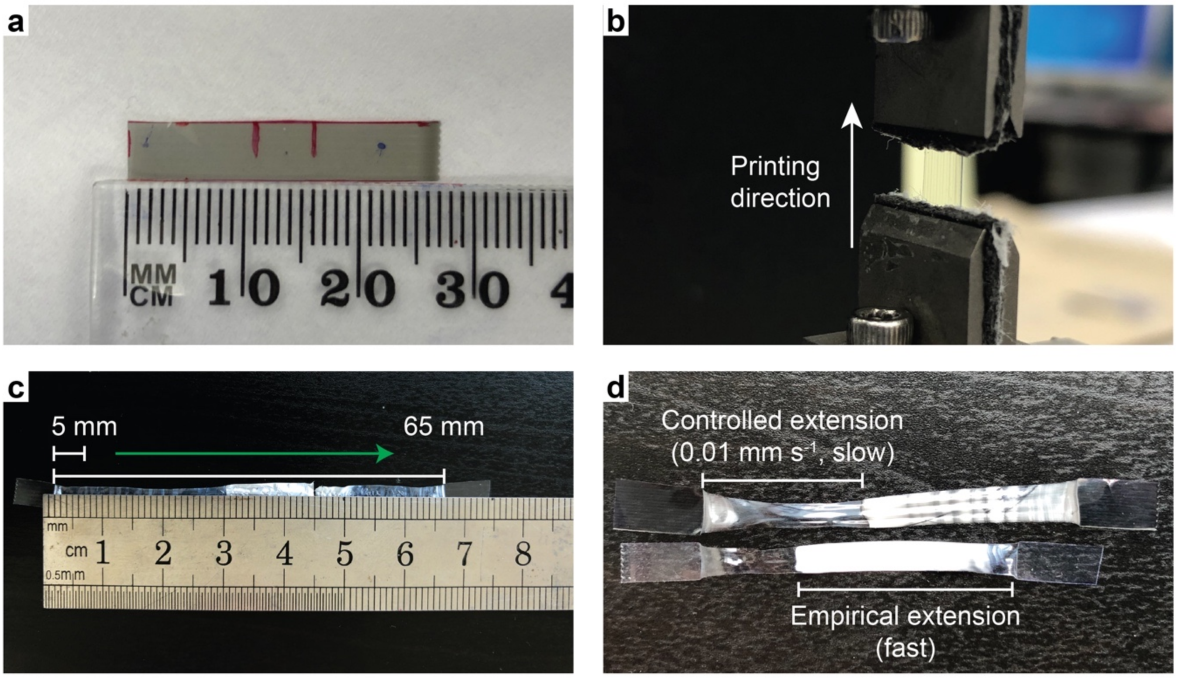

Supplementary Fig. 13: The experimental layout of the mechanical testing. **a** Photograph of the geometry of the tested samples, with markings representing the position of the grips. **b** Photograph showing the sample inserted into the grips prior to extension. **c** Photograph showing the extent of extension required to break the SEA extrusion printed pristine PVDF-TrFE co-polymer film. **d** Photograph showing the dependence of transparency on the extension rate, shown for the SEA extrusion printed $Ti_3C_2T_x$/PVDF-TrFE (0.10 wt%) film (top) and SEA extrusion printed pristine PVDF-TrFE co-polymer film (bottom).

Supplementary Table 1: Experimentally determined width (w) and thickness (t) for each sample measured during the tensile mechanical testing. The S values designate the sample number.

	Width (w, mm)			Thickness (t, μm)		
	S1	S2	S3	S1	S2	S3
$Ti_3C_2T_x$/PVDF-TrFE (0.00 wt%)	4.3	5.0	4.9	53	53	59
$Ti_3C_2T_x$/PVDF-TrFE (0.02 wt%)	5.2	5.0	5.2	54	49	51
$Ti_3C_2T_x$/PVDF-TrFE (0.10 wt%)	5.1	5.1	5.2	45	46	47
$Ti_3C_2T_x$/PVDF-TrFE (0.20 wt%)	4.8	5.1	5.0	36	37	37
$Ti_3C_2T_x$/PVDF-TrFE (0.50 wt%)	5.0	5.0	5.0	35	35	35

The tensile strain (γ_t) was calculated from the data obtained during tests following Equation S1, where L is the displacement and L_0 is the distance between grips at the beginning of the test (5 mm):

$$\gamma_t = L/L_0 \tag{S1}$$

The tensile stress (σ_t) was calculated from the data obtained during tests, using the cross-sectional area of the sample (A_{cs}), following Equation S2:

$$\sigma_t = \frac{F}{A_{cs}} = \frac{F}{t \times w} \tag{S2}$$

Here, F is the measured force, t is the thickness of the sample, and w is the width of the sample (cut to approximately 5 mm).

Due to the low displacement limit of the instrument, the strain at break was approximated via empirical measurements (Supplementary Fig. 13c), namely extending by hand. While these tests were merely representative, the samples were found to stretch to at least 65 mm prior to breaking, corresponding to 1,300% of the L_0 (5 mm). Additionally, the final transparency in

the extended regions was observed to be higher when the extension rate was slower (Supplementary Fig. 13d).

Optical properties

The optical properties of the extrusion printed $Ti_3C_2T_x$/PVDF-TrFE films were characterized by ultraviolet, visible and near-infrared (UV-vis-NIR) spectrophotometry (Lambda 950, Perkin Elmer). Spectra were obtained in the visible wavelength range (between 380 nm and 780 nm) with a step size of 5 nm. The incident light (T_1) was captured with no sample in place. The samples were then placed into a custom-made holder and secured to the entry port of a 150 mm diameter integrating sphere to capture all non-absorbed light (T_2). The total transmittance (T_t) of the sample was then taken as the fraction between T_2 and T_1 (Supplementary Fig. 14a). The scattered light intensity in the instrument (T_3) was measured without the sample, using a light trap directly in the path of the beam (with diameter corresponding to 2° of the integrating sphere). Finally, the sample scatter (T_4) was measured with the light trap in place and the sample covering the entry port of the integrating sphere. The diffuse transmittance (T_d) was calculated following Equation S3 (Supplementary Fig. 14b) and the haze was calculated in accordance with the ASTM D1003 standard,[26] using Equation S4 (Supplementary Fig. 14c).

$$T_d = (T_4 - T_3 T_t)/T_1 \tag{S3}$$

$$Haze\ (\%) = T_d/T_t \times 100 \tag{S4}$$

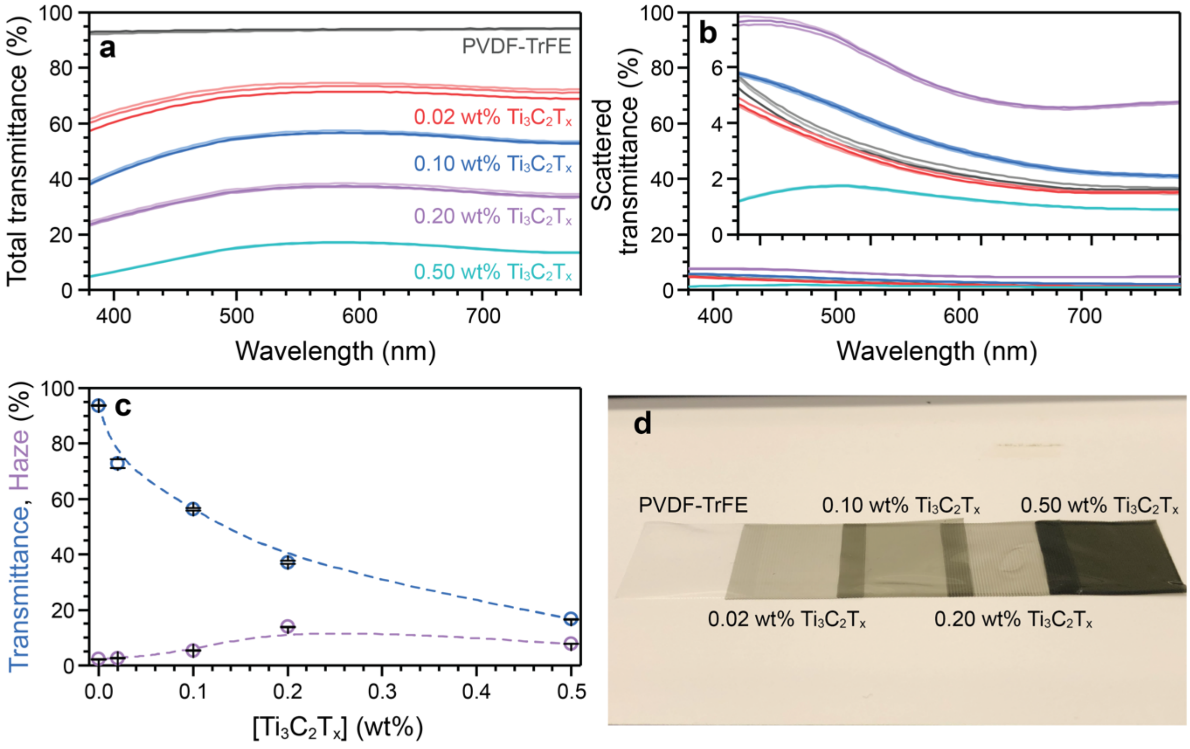

Supplementary Fig. 14: Optical properties of SEA extrusion printed $Ti_3C_2T_x$/PVDF-TrFE (0.00 wt%, 0.02 wt%, 0.10 wt%, 0.20 wt% and 0.50 wt%) films. **a** The total light transmittance (T_t). **b** The scattered light transmittance (T_d). **c** The transmittance and haze as a function of $Ti_3C_2T_x$ nanosheet concentration. **d** Side by side photograph showing the color and transmittance of the films. The error bars in **c** represent the mean ± SD.

As expected, the transmittance was found to decrease as the $Ti_3C_2T_x$ nanosheet concentration increased, from 94% for pristine PVDF-TrFE co-polymer to 20% for the $Ti_3C_2T_x$/PVDF-TrFE (0.50 wt%) film (Supplementary Fig. 14a,c,d). Surprisingly, the addition of $Ti_3C_2T_x$ nanosheets did not significantly increase the scattered light intensity and haze (Supplementary Fig. 14b,c, Fig. 3e), with the maximum obtained haze found in the $Ti_3C_2T_x$/PVDF-TrFE (0.20 wt%) film to be 13.9%, compared to the pristine PVDF-TrFE co-polymer film (2.2%). In fact, the scattered light transmittance (Supplementary Fig. 14b) was found to be lower at all wavelengths in the $Ti_3C_2T_x$/PVDF-TrFE (0.02 wt% and 0.50 wt%) films, compared to the pristine PVDF-TrFE film, suggesting the increase in haze with the incorporation of $Ti_3C_2T_x$ nanosheets was largely governed by the decrease in the transmittance, as opposed to the scattering from aggregated $Ti_3C_2T_x$ nanosheets.

Raman analysis

The evolution of the Raman spectra when the $Ti_3C_2T_x$ nanosheets are added to the PVDF-TrFE co-polymer reveals a clear suppression of out-of-plane vibrational modes occurring in $Ti_3C_2T_x$/PVDF-TrFE films. These modes occurring at 700 – 720 cm^{-1} and 200 cm^{-1} correspond to the out-of-plane A_{1g} vibrational modes for oxygen functional groups bound to the $Ti_3C_2T_x$ lattice, whereas the peaks between 250 cm^{-1} and 700 cm^{-1} all correspond to in-plane E_g vibrational modes.[27] Notably, the A_{1g} modes at 700 – 720 cm^{-1} disappear almost completely, even in the $Ti_3C_2T_x$/PVDF-TrFE (0.50 wt%) films, with no difference between solvent-cast and extrusion printed films (Fig. 4a). In contrast, the intensity of the higher energy A_{1g} mode at 200 cm^{-1} appears unchanged or even have an increased intensity relative to the main E_{2g} modes (Fig. 4a). While this contrast in intensity change appears anomalous, it supports the data for well exfoliated flakes in literature.[27] Here, it should be noted that the Raman spectrum of the $Ti_3C_2T_x$ nanosheets was attained by drop-casting $Ti_3C_2T_x$ nanosheets in DMF on a silicon wafer, likely resulting in restacking and stronger A_{1g} modes. The absence, or weak intensity, of these A_{1g} modes in the $Ti_3C_2T_x$/PVDF-TrFE films therefore implies two key points, (1) that the PVDF-TrFE co-polymer is an excellent stabilizing agent for the $Ti_3C_2T_x$ nanosheets as there is no evidence of restacking; and (2) there is a strong binding between the PVDF-TrFE co-polymer and $Ti_3C_2T_x$ nanosheets (and subsequent polymer densification) such that the A_{1g} modes are even further weakened and shifted.[28] These results confirm the strong electrostatic binding as predicted by MD simulations (Fig. 2a).

Raman mapping of the surface of the $Ti_3C_2T_x$/PVDF-TrFE films showed a significantly variable response in the I_β/I_γ ratio (Supplementary Fig. 15). This variation was most noticeable for the SEA extrusion printed $Ti_3C_2T_x$/PVDF-TrFE (0.02 wt%) film and decreased with an increased $Ti_3C_2T_x$ nanosheet loading up to 0.50 wt%, where the sample presented a

homogenous ratio. This improvement in sample homogeneity at higher $Ti_3C_2T_x$ nanosheet loadings is hypothesized to be due to the discrepancy in the state of the PVDF-TrFE co-polymer when it is bound to the $Ti_3C_2T_x$ nanosheet basal plane. At higher $Ti_3C_2T_x$ nanosheet loadings, we propose a high proportion of the PVDF-TrFE co-polymer is within the electrostatic sphere of influence (between 1 nm and 10 nm) of the $Ti_3C_2T_x$, thus presenting a homogenous I_β/I_γ.[29] The data from these maps was averaged and used for describing the average sample spectra and I_β/I_γ (Fig. 4a,b). The solvent cast $Ti_3C_2T_x$/PVDF-TrFE film shows a higher variation in I_β/I_γ (Supplementary Fig. 14f) which is attributed to the lack of homogenization of 2D materials by the extrusion printing process.

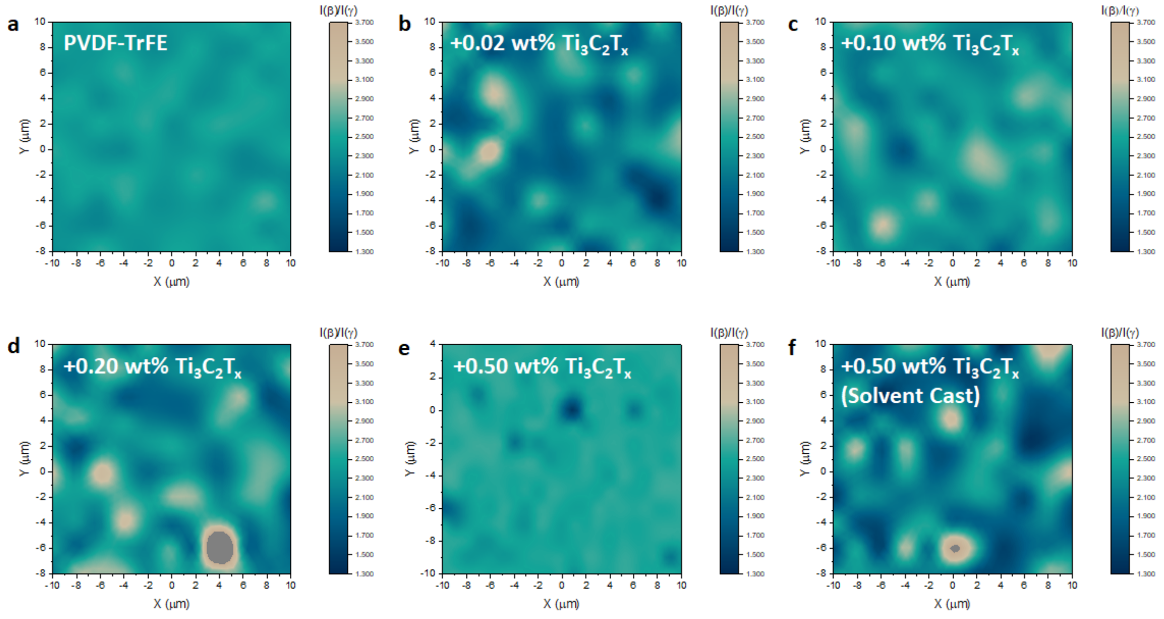

Supplementary Fig. 15: The confocal Raman microscopy maps of the surface of the films, showing the intensity ratio (I_β/I_γ) between the β phase peak (842 cm^{-1}) and γ phase peak (811 cm^{-1}). **a** The SEA extrusion printed pristine PVDF-TrFE co-polymer film. **b** The SEA extrusion printed $Ti_3C_2T_x$/PVDF-TrFE (0.02 wt%) film. **c** The SEA extrusion printed $Ti_3C_2T_x$/PVDF-TrFE (0.10 wt%) film. **d** The SEA extrusion printed $Ti_3C_2T_x$/PVDF-TrFE (0.20 wt%) film. **e** The SEA extrusion printed $Ti_3C_2T_x$/PVDF-TrFE (0.50 wt%) film. **f** The solvent cast $Ti_3C_2T_x$/PVDF-TrFE (0.50 wt%) film.

Attenuated total reflection Fourier transform infrared (ATR-FTIR) spectroscopy

ATR-FTIR spectroscopy was performed on the samples using an ALPHA II spectrometer (Bruker). Absorbance spectra were collected by taking an average of 128 individual scans at a resolution of 1 cm^{-1}, between 600 cm^{-1} and 4000 cm^{-1}.

ATR-FTIR spectroscopy was used to estimate the fraction of phases in the SEA extrusion printed $Ti_3C_2T_x$/PVDF-TrFE films, for $Ti_3C_2T_x$ nanosheet concentrations at 0.00 wt%, 0.02 wt%, 0.10 wt%, 0.20 wt% and 0.50 wt% (Supplementary Fig. 16a). The peak commonly attributed to the α phase (766 cm^{-1}) was not distinctly visible in all the measured spectra, suggesting the low fraction of the α phase in the bulk of the samples.[17] Notably, as was determined by Raman microscopy (Fig. 4d,e), the α phase was present in close proximity to the $Ti_3C_2T_x$ nanosheet surface; however, the ATR-FTIR spectra suggested a low fraction of the α phase in the bulk of the $Ti_3C_2T_x$/PVDF-TrFE films. The peak at 840 cm^{-1}, indicative of the total electroactive phase (consisting of β and γ phases, denoted as β+γ), was present in all measured samples.[16] The separate peaks γ phase (1235 cm^{-1}) and β phase (1290 cm^{-1}) were both observed, confirming the presence of both electroactive phases (β+γ); however, the separate peaks could not be deconvoluted as the γ phase peak was present as a shoulder.

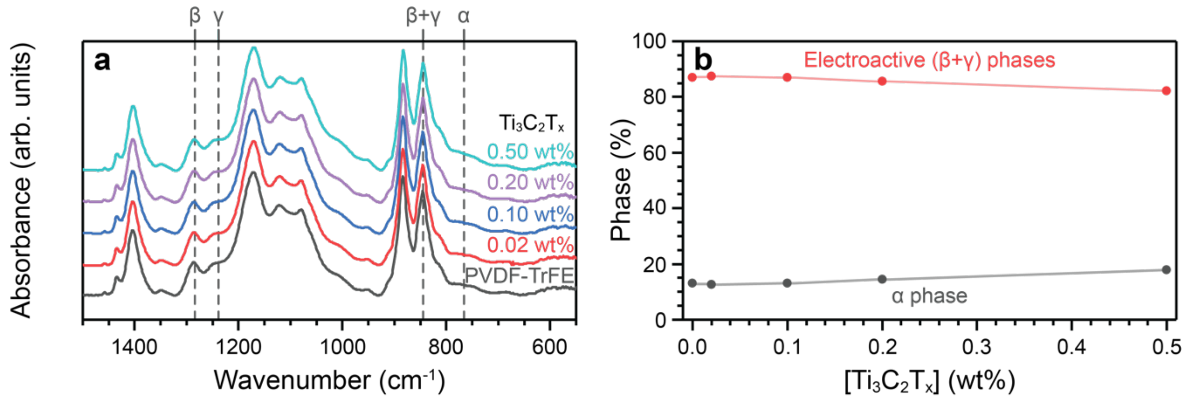

Fig. S16: ATR-FTIR spectroscopy characterization of SEA extrusion printed $Ti_3C_2T_x$/PVDF-TrFE (0.00 wt%, 0.02 wt%, 0.10 wt%, 0.20 wt% and 0.50 wt%) films. **a** The FTIR spectra, offset for clarity. **b** The relative fractions of the electroactive (β+ γ) phases and the α phase as a function of $Ti_3C_2T_x$ concentration.

The total electroactive phase fraction (F_{ea}) was calculated using ATR-FTIR data by Equation S5:

$$F_{ea}(\%) = \frac{I_{ea}}{\left({K_{ea}}/{K_{\alpha}}\right) I_{\alpha} + I_{ea}} \quad \text{(S5)}$$

Here, I_{ea} is the intensity of β+γ peak, I_{α} is the intensity of α peak, K_{α} and K_{ea} are the absorption coefficients for the peaks at 766 cm^{-1} and 840 cm^{-1}, with values of 6.1 x 10^4 cm^2 mol^{-1} and 7.7 x 10^4 cm^2 mol^{-1}, respectively.[16] The F_{ea} of the pristine PVDF-TrFE co-polymer film was 87.0% (Supplementary Fig. 16b), significantly higher relative to the pristine PVDF-TrFE films SEA extrusion printed from a solvent mixture of DMF and acetone (40:60 vol%).[10] The highest F_{ea} value was observed at 87.5% for the $Ti_3C_2T_x$/PVDF-TrFE (0.02 wt%) film, although this value exhibited little deviation from that of the pristine PVDF-TrFE film. Notably, at $Ti_3C_2T_x$/PVDF-TrFE (0.50 wt%), the F_{ea} was found to decrease to 82.0%, consistent with the local α phase formation in the PVDF-TrFE on the surface of the $Ti_3C_2T_x$ nanosheets (Fig. 4e).

X-ray powder diffractometry (XRD)

XRD spectra were obtained for the SEA extrusion printed $Ti_3C_2T_x$/PVDF-TrFE films (0.00 wt%, 0.02 wt%, 0.10 wt%, 0.20 wt%, 0.50 wt%) using Bragg–Brentano geometry (D8 Advance, Bruker) using Cu-Kα radiation (λ = 1.54060 Å). The Bragg angle, 2θ, was varied between 5° and 70° with a step size of 0.02° and 1 s per step, with the sample rotated at 15 rpm. The films were placed on silicon low background holders for the measurements.

The phase distribution in the SEA extrusion printed films was analyzed with XRD (Supplementary Fig. 17). Two main peaks were visible in the spectra. The broad peak at 18.1° was attributed to the (110/200) paraelectric γ phase reflection. The sharp peak at 20.2° corresponds to the (110/200) ferroelectric β phase reflection in PVDF-TrFE.[30] Due to the low

concentration of $Ti_3C_2T_x$ nanosheets, no peaks were observed for the additive (Supplementary Fig. 1a) in the spectra. Additional peaks were found at 35.0° and 40.8°, attributed to the (001) ferroelectric phase reflection and (111/201), (400/220) ferroelectric phase reflections, respectively, confirming the primary presence of the β phase.[31,32]

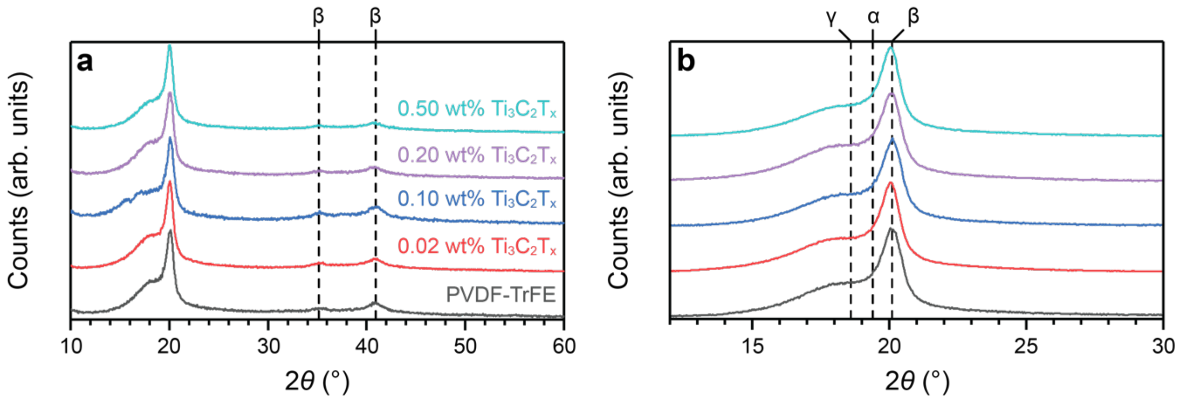

Fig. S17: XRD spectra of the SEA extrusion printed $Ti_3C_2T_x$/PVDF-TrFE films. **a** Survey scan. **b** Phase fingerprint region. Spectra offset for clarity.

The primary fingerprint region (Supplementary Fig. 17b) region was further deconvoluted to investigate the distribution of phase fractions (Supplementary Fig. 18). The region required four peaks to ensure the correct fit. The strongest peak was assigned to the β phase (blue), the broad second peak was assigned to the γ phase (purple), the third peak was attributed to the α phase (yellow) and the final peak corresponded to the amorphous regions of the polymer (gray).

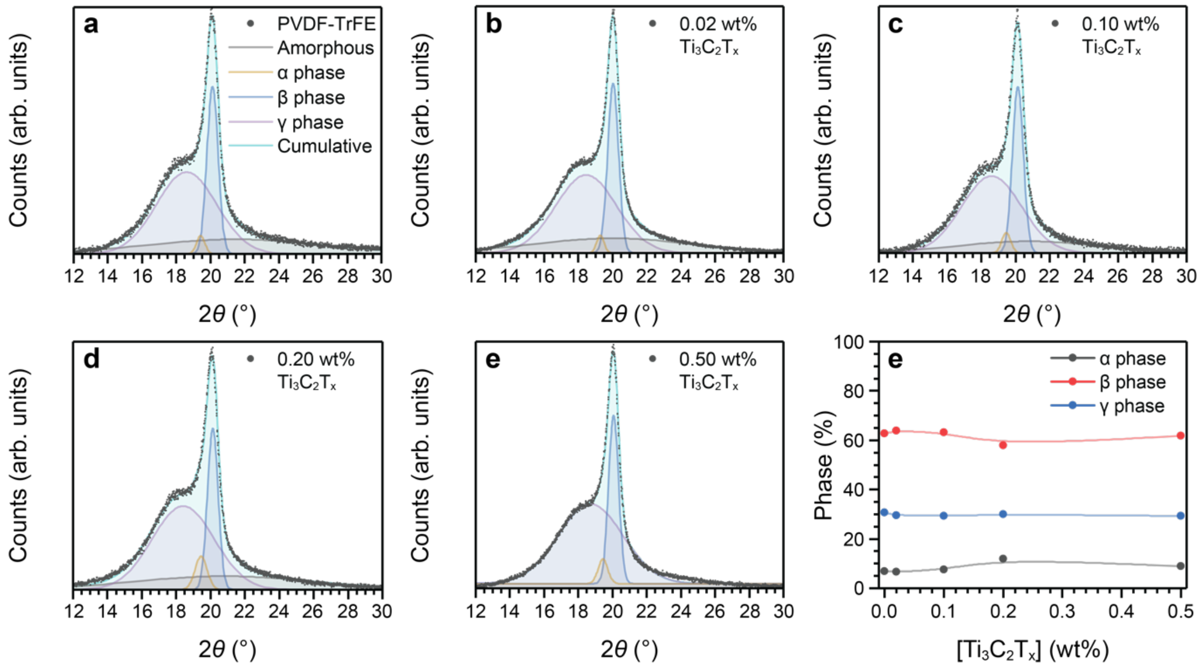

Supplementary Fig. 18: XRD deconvolution for the phases in SEA extrusion printed films. **a** Pristine PVDF-TrFE copolymer. **b** $Ti_3C_2T_x$/PVDF-TrFE (0.02 wt%). **c** $Ti_3C_2T_x$/PVDF-TrFE (0.10 wt%). **d** $Ti_3C_2T_x$/PVDF-TrFE (0.20 wt%). **e** $Ti_3C_2T_x$/PVDF-TrFE (0.50 wt%). **f** Phase fractions determined from XRD as a function of $Ti_3C_2T_x$ nanosheet concentration.

The deconvoluted XRD spectra (Supplementary Fig. S18a-e) were used to calculate the phase fractions (Supplementary Fig. 18f) within the PVDF-TrFE co-polymer in the SEA extrusion printed $Ti_3C_2T_x$/PVDF-TrFE (0.00 wt%, 0.02 wt%, 0.10 wt%, 0.20 wt%, 0.50 wt%) films from the intensities for the respective peaks following Equations S6a-c:

$$F_\alpha = \frac{I_\alpha}{I_\alpha + I_\beta + I_\gamma} \tag{S6a}$$

$$F_\beta = \frac{I_\beta}{I_\alpha + I_\beta + I_\gamma} \tag{S6b}$$

$$F_\gamma = \frac{I_\gamma}{I_\alpha + I_\beta + I_\gamma} \tag{S6c}$$

Here, I_α, I_β and I_γ correspond to the intensities for the peaks found at 19.4°, 20.2° and 18.1°, respectively. The phase distributions were found to correlate well with the data obtained from Raman spectroscopy (Fig. 4b) and FTIR spectroscopy (Supplementary Fig. 16b). In particular, the F_{ea} calculated from the FTIR spectra for the $Ti_3C_2T_x$/PVDF-TrFE films at $Ti_3C_2T_x$

nanosheet concentrations below 0.50 wt% (87%) matched closely with the sum of F_β and F_γ calculated from the deconvoluted XRD (between 85% and 90%). Furthermore, the Raman spectroscopy has suggested the primary phases in the bulk are the β and γ phases (Fig. 4a), with the β phase as the primary component, which is in close agreement with the XRD data.

Differential scanning calorimetry (DSC)

The crystallinity of the PVDF-TrFE co-polymer in the SEA extrusion printed $Ti_3C_2T_x$/PVDF-TrFE (0.00 wt%, 0.02 wt%, 0.10 wt%, 0.20 wt%, 0.50 wt%) films was measured by DSC (STA 5000, Perkin Elmer). The samples were heated from 25 °C in a ceramic sample pan (N5200040, Perkin Elmer) to 200 °C at a rate of 10 °C min^{-1} under a nitrogen flow at 20 mL min^{-1} (99.999% purity, BOC).

The DSC thermograms (Supplementary Fig. 19) showed two endothermic peaks for all analyzed samples, centered at approximately 105 °C and 142 °C.[10] The peak at 105 °C corresponded to the ferroelectric to paraelectric transition (Curie temperature, T_c), whereby the samples exhibit piezoelectric properties below the T_c and lose polarization above the T_c.[33] The primary peak at 142 °C corresponds to the melting of the polymer (T_m), with the enthalpy (ΔH_m) correlating to the crystallinity (χ_c) following Equation S7, where the enthalpy of melting for completely crystalline PVDF-TrFE (ΔH_0) is given as 45 J g^{-1}, shown in Fig. 4c of the main text.[34,36]

$$\chi_c = \frac{\Delta H_m}{\Delta H_0} \tag{S7}$$

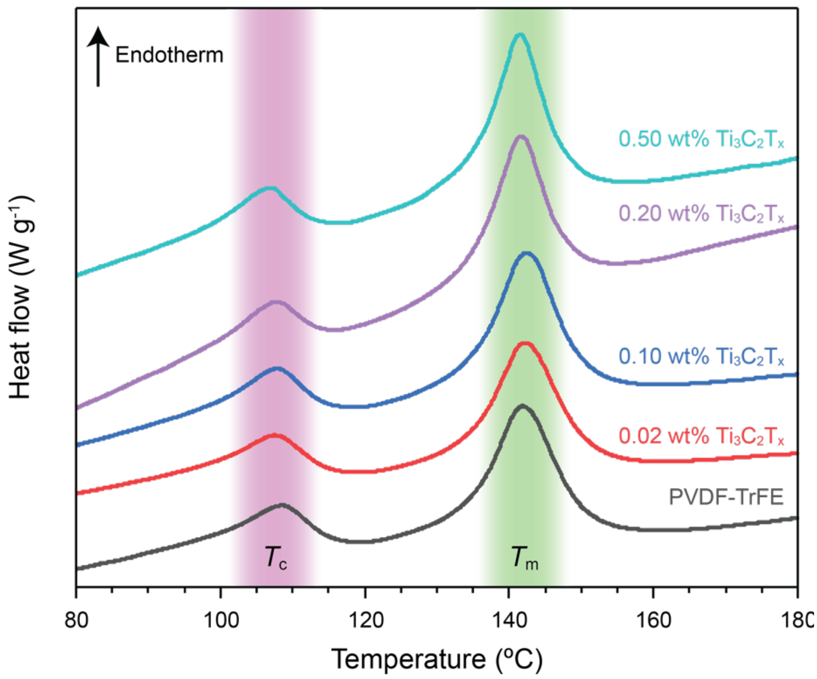

Supplementary Fig. 19: DSC thermograms of the SEA extrusion printed $Ti_3C_2T_x$/PVDF-TrFE films, offset for clarity.

Piezoresponse force microscopy (PFM) of $Ti_3C_2T_x$/PVDF-TrFE films

PFM was carried out on the $Ti_3C_2T_x$ nanosheets, SEA extrusion printed $Ti_3C_2T_x$/PVDF-TrFE (0.00 wt%, 0.02 wt%, 0.10 wt%, 0.20 wt% and 0.50 wt%) films and solvent cast $Ti_3C_2T_x$/PVDF-TrFE (0.00 wt% and 0.50 wt%) films in order to probe the trends in the out-of-plane polarization at the nanoscale. An atomic force microscope (Cypher ES, Oxford Instruments) with a high voltage accessory, equipped with a solid platinum cantilever (12Pt400A, Rocky Mountain Nanotechnology) was used for this testing.

The PFM of the $Ti_3C_2T_x$ nanosheets was carried out in dual AC resonance tracking (DART) mode at a bias of 1 V to confirm the absence of out-of-plane piezoelectricity. The scans were taken with 512 pixels per line and frequency at 0.5 Hz. To prepare the samples, the $Ti_3C_2T_x$ nanosheets were cast from solvent (DMF) onto gold (Au) coated silicon (Si) wafers and dried under vacuum. The DART-PFM scans were taken at three scales, 5 µm (Supplementary Fig. 20a, d, g), 2 µm (Supplementary Fig. 20b, e, h), and 1 µm (Supplementary Fig. 20c, f, i and Fig. 5a). No discernible difference between the $Ti_3C_2T_x$ nanosheets (visible in the topography trace, Supplementary Fig. 20a-c) and the Si substrate was found in the phase (Supplementary Fig. 20d-f) or amplitude (Supplementary Fig. 20g-i) traces. Therefore, the $Ti_3C_2T_x$ nanosheets were not observed to exhibit out-of-plane polarization and assumed as a non-piezoelectric additive in these experiments.

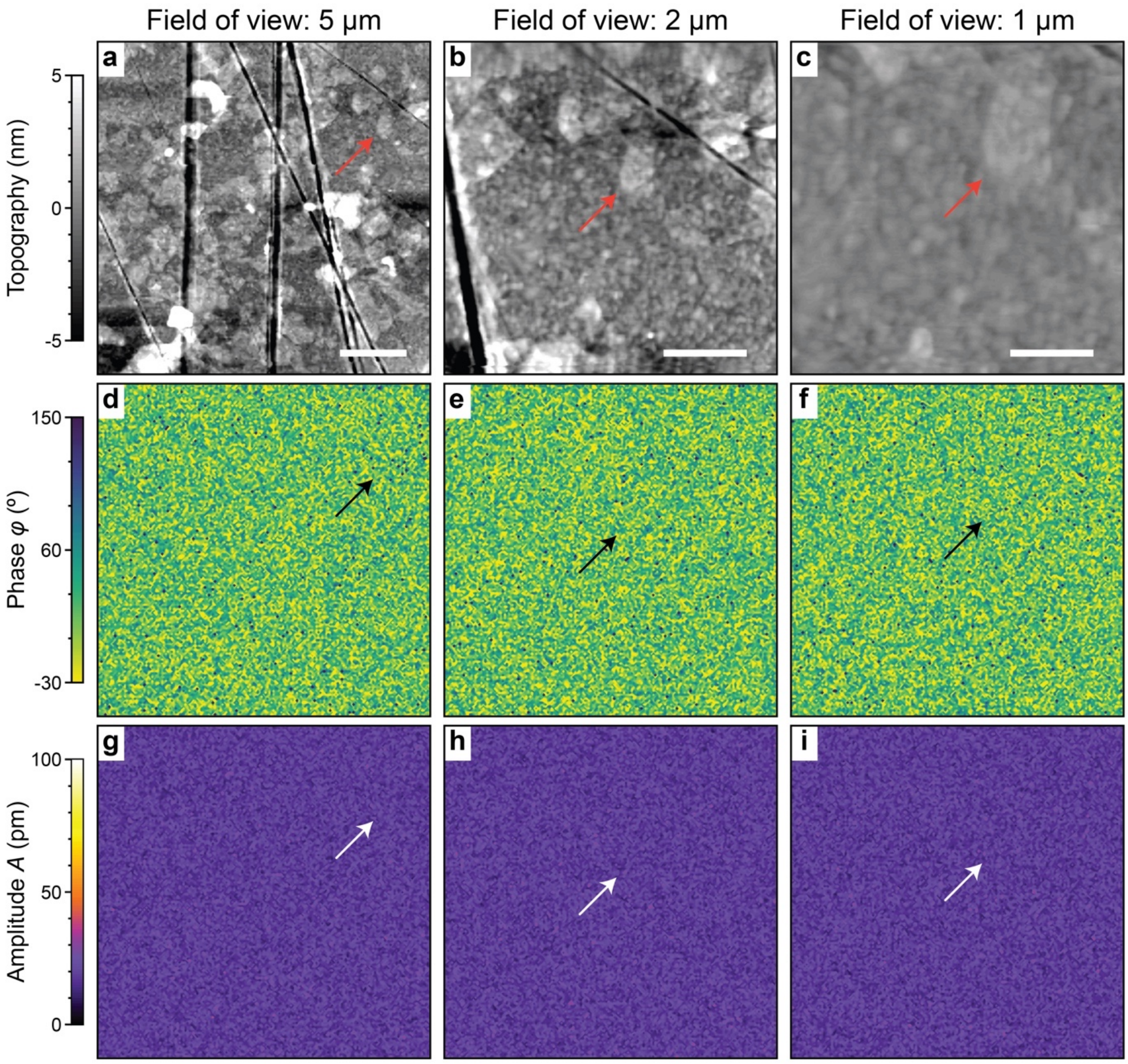

Supplementary Fig. 20: DART-PFM maps of $Ti_3C_2T_x$ nanosheets adsorbed on a gold (Au)-coated silicon (Si) wafer. **a-c** The topography traces. **d-f** The piezoelectric phase traces. **g-i** The piezoelectric amplitude traces. **a, d, g** Field of view corresponding to 5 µm. Scale bar represents 1 µm. **b, e, h** Field of view corresponding to 2 µm. Scale bar represents 500 nm. **c, f, i** Field of view corresponding to 1 µm. Scale bar represents 250 nm. The arrow in each panel points to the location of the same $Ti_3C_2T_x$ nanosheet.

The PFM of the $Ti_3C_2T_x$/PVDF-TrFE films was carried out in lithography mode,[10] whereby a bias was applied to individual regions, monitoring the piezoelectric response through the converse piezoelectric effect ($\gamma_3 = d_{33}E_3$, whereby γ_3 is the out-of-plane strain, d_{33} is the piezoelectric coefficient and E_3 is the out of plane electric field).[16,36,37] The lithography mode was chosen to obtain data below the poling field, where typical ferroelectric hysteresis loops cannot be formed, such that the poling state of the material would be minimally altered.[10] The

applied voltage was between -20 V and +20 V, in increments of 2 V (Supplementary Fig. 21), imaged over an area with lateral dimensions at 5 μm and 256 lines per scan, corresponding to a resolution of approximately 19 nm per pixel, with a scan rate of 0.2 Hz.

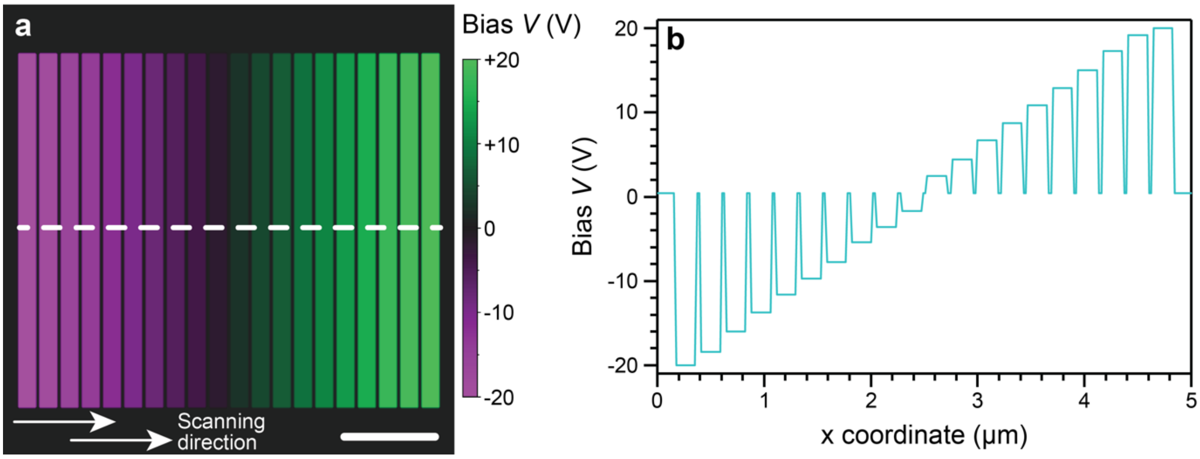

Supplementary Fig. 21: Applied bias characteristics during the PFM measurements on the $Ti_3C_2T_x$/PVDF-TrFE films. **a** Map of applied bias. Scale bar represents 1 μm. **b** Bias as a function of the x coordinate for a single line in the scan direction, which is signified by the dashed line in **a**.

Each voltage was applied to the sample in a rectangular pattern, with the length at 200 pixels and width at 10 pixels, such that each scanned line contains the PFM data for all applied voltages (Supplementary Fig. 21b). The measured data contained the topography, piezoelectric amplitude (A) and piezoelectric phase (φ). The data was obtained both for SEA extrusion printed (Supplementary Fig. 22) and solvent-cast (Supplementary Fig. 23) $Ti_3C_2T_x$/PVDF-TrFE films.

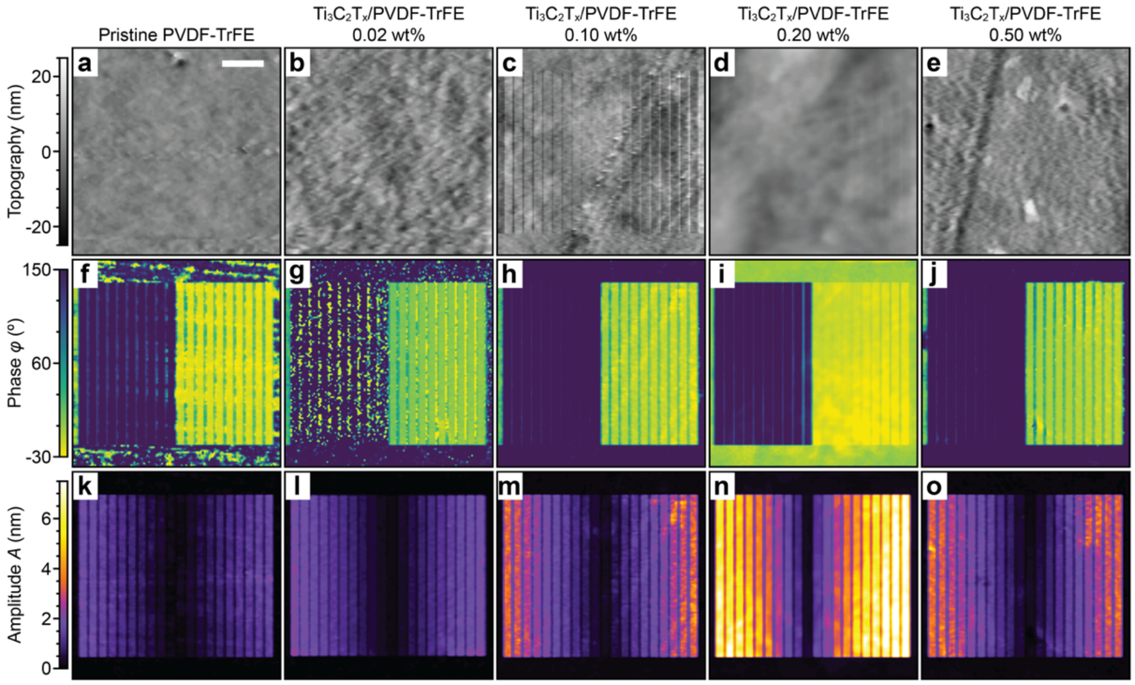

Supplementary Fig. 22: Raw PFM data obtained for the SEA extrusion printed $Ti_3C_2T_x$/PVDF-TrFE films. **a-e** The topography. **f-j** The piezoelectric phase (φ). **k-o** The piezoelectric amplitude (A). The data was obtained for **a, f, k** pristine PVDF-TrFE (0.00 wt% $Ti_3C_2T_x$), **b, g, l** $Ti_3C_2T_x$/PVDF-TrFE (0.02 wt%), **c, h, m** $Ti_3C_2T_x$/PVDF-TrFE (0.10 wt%), **d, i, n** $Ti_3C_2T_x$/PVDF-TrFE (0.20 wt%), **e, j, o** $Ti_3C_2T_x$/PVDF-TrFE (0.50 wt%). The scale bar represents 1 µm.

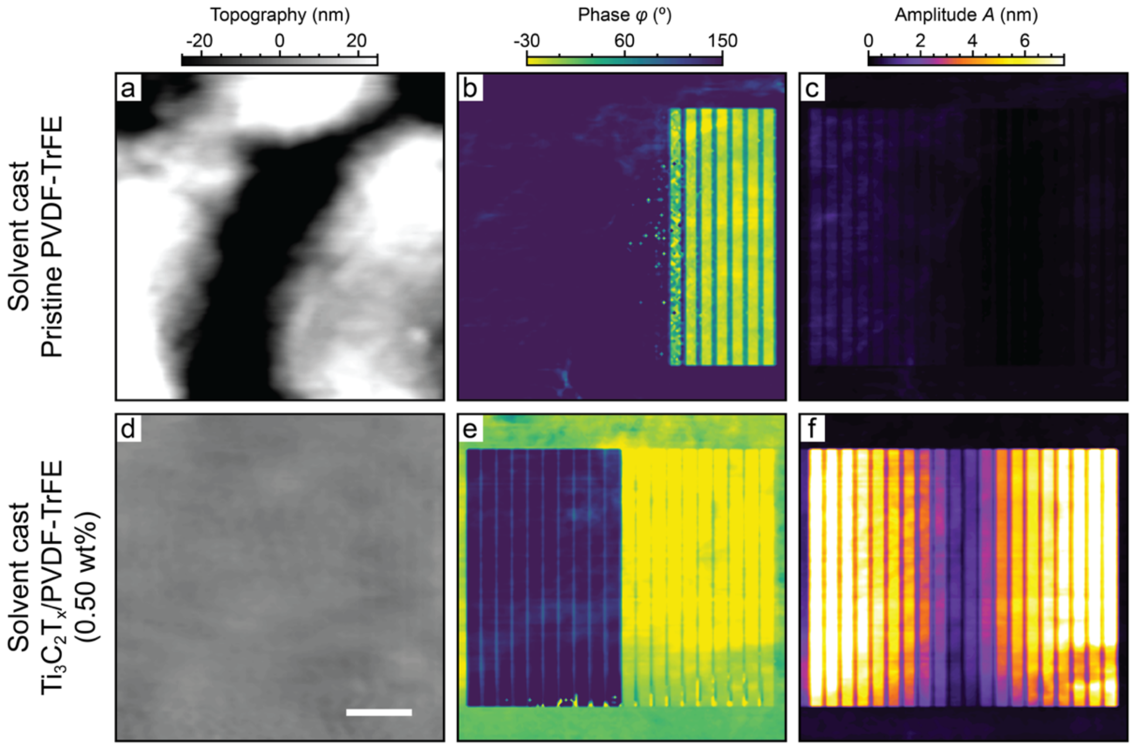

Supplementary Fig. 23: Raw PFM data obtained for the solvent cast **a-c** pristine PVDF-TrFE and **d-f** $Ti_3C_2T_x$/PVDF-TrFE (0.50 wt%) films. **a, d** The topography. **b, e** The piezoelectric phase (φ). **c, f** The piezoelectric amplitude (A). The scale bar represents 1 µm.

The A and φ data (Supplementary Fig. 22f-o, Supplementary Fig. 23b, c, e, f) was processed using purpose-built Matlab code, which undertook pixel-by-pixel operations (Supplementary Fig. 24) to form the data obtained in Fig. 5b by multiplying the A signal (Supplementary Fig. 24b) by the cosine of the φ signal (Supplementary Fig. 24a,c) and dividing by the Q factor of the cantilever (Q_f) for each applied bias (Supplementary Fig. 24d). The Q_f was measured in the tuning stage directly prior to the measurement (Supplementary Table 2). To calculate the effective d_{33}, the $Acos(\varphi)/Q_f$ data was separated by the applied bias and averaged, obtaining a plot for $Acos(\varphi)/Q_f$ as a function of the applied bias for the samples (Supplementary Fig. 24d).

Supplementary Table 2: Experimentally determined Q factor (Q_f) for each PFM scan.

	Q factor (Q_f, arb. units)	
	SEA extrusion printed	Solvent-cast
Pristine PVDF-TrFE (0.00 wt% $Ti_3C_2T_x$)	39.389	41.244
$Ti_3C_2T_x$/PVDF-TrFE (0.02 wt%)	36.083	-
$Ti_3C_2T_x$/PVDF-TrFE (0.10 wt%)	37.185	-
$Ti_3C_2T_x$/PVDF-TrFE (0.20 wt%)	73.754	-
$Ti_3C_2T_x$/PVDF-TrFE (0.50 wt%)	31.188	75.479

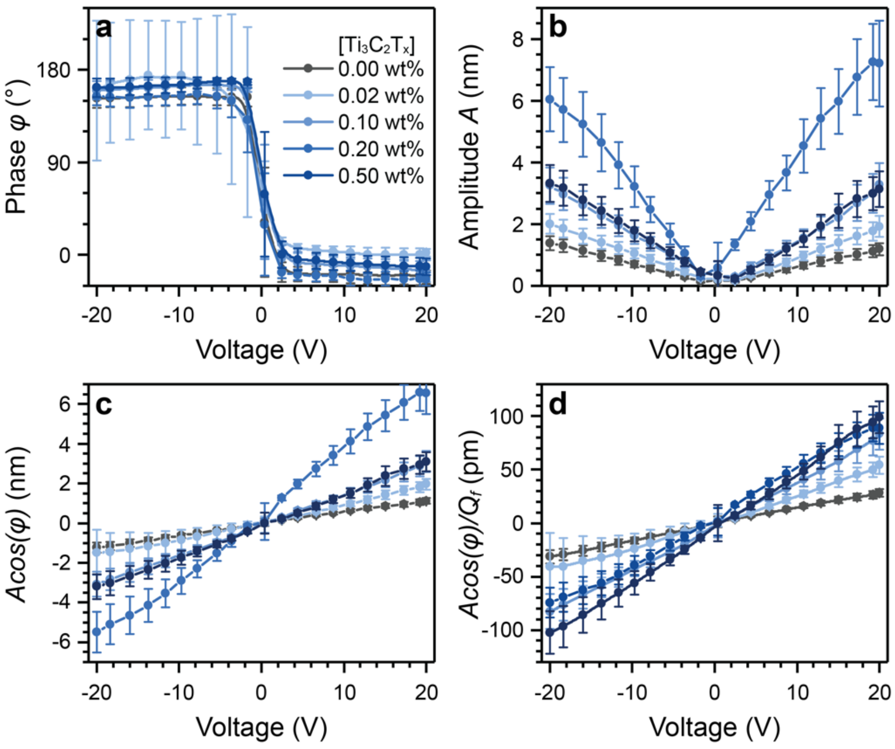

Supplementary Fig. 24: Processed PFM results as a function of the input bias (*V*) for the SEA extrusion printed $Ti_3C_2T_x$/PVDF-TrFE films. **a** The phase (φ). **b** The amplitude (A). **c** The $Acos(\varphi)$. **d** The $Acos(\varphi)/Q_f$. The error bars represent the mean ± SD.

In the converse piezoelectric effect, the d_{33} is given as shown in Equation S8a:[37]

$$d_{33} = \left(\frac{\partial \gamma_3}{\partial E_3}\right)^{\sigma} \tag{S8a}$$

Here, the superscript σ denotes constant stress. In order to minimize the stress on the sample, the cantilever is required to possess a sufficiently low spring constant, in this case approximately 0.3 N m^{-1}. It should be noted, however, that the cantilever will nonetheless apply stress to the sample, therefore restricting the expansion in the measured material and providing an underestimate to the calculated d_{33}. The out-of-plane strain is given as $\gamma_3 = L/L_0$, whereby L is the magnitude of the expansion or contraction, and L_0 is the material thickness. In PFM, L corresponds to the normalized amplitude, shown in Equation S8b:

$$L = \frac{Acos(\varphi)}{Q_f} \tag{S8b}$$

Therefore, the out-of-plane strain then takes on the form shown in Equation S8c:

$$\gamma_3 = \frac{Acos(\varphi)}{Q_f L_0} \qquad \text{(S8c)}$$

Moreover, the E_3 is given as the *V* applied per unit distance. In the case of PFM, as the material expands upon applied *V*, the expansion should include the distance of expansion, shown in Equation S8d:

$$E_3 = \frac{V}{L + L_0} \qquad \text{(S8d)}$$

Hence, substituting Equation 8b for *L*, the expression becomes as shown in Equation S8e:

$$E_3 = \frac{V}{\left(Acos(\varphi)/Q_f\right) + L_0} \qquad \text{(S8e)}$$

Finally, substituting Equation S8c and Equation S8e into Equation S8a, it takes on the form as shown in Equation S8f:

$$d_{33} = \frac{Acos(\varphi)}{Q_f V} + \frac{\left(Acos(\varphi)\right)^2}{Q_f^2 L_0 V} \qquad \text{(S8f)}$$

Notably, this expression still does not account for the stress applied to the sample by the cantilever, however it becomes a more accurate equation to obtain the d_{33}. Nonetheless, when a 10 nm *A* (1.00×10^{-8} m) is observed at 180° φ (corresponding to $cos(\varphi) = 1$) in a sample with 40 µm (4.00×10^{-5} m) L_0 under 20 V applied bias and Q_f at 30, the additional term corresponds to an increase in the effective d_{33} of 1.39×10^{-4} pm V^{-1}, a minute increase relative to an effective d_{33} at 16.67 pm V^{-1}. Therefore, for these experiments, the effective d_{33} was taken as $Acos(\varphi)/Q_f V$.

As the applied *V* was below the poling voltage (maximum *V* at 20 V, minimum thickness at 37 µm, corresponding to a maximum $E_3 = 0.54$ MV m^{-1}, minimum poling E_3 at 50 MV m^{-1}),[18] the slope of the plot for $Acos(\varphi)/Q_f$ as a function of *V* was expected to be linear for all samples.

Indeed, the data shown in Supplementary Fig. 24d was found to be linear over the measured range, taking into account the deviation over the measured scan area. The data was fit with a linear trendline for each sample, whereby the slope of the trendline, accounting for the error in each sample, was the effective d_{33} value.

The accuracy of the nanoscale polarization measurements via PFM has been widely debated in recent literature, demonstrating the values can underrepresent or overrepresent the macroscale d_{33} both due to an empirical calculation methodology and the localized measurement approach.[36,38] Nonetheless, these PFM experiments are able to show trends of the d_{33} in samples with similar composition, as has been demonstrated here. Notably, the most accurate methodology is to utilize a single cantilever, as has been undertaken in these experiments. The utilization of multiple cantilevers has the potential to vary in the spring constant and therefore dampen the amplitude signal, subsequently changing the observed trends.

Macroscale energy harvesting characteristics

Piezoelectric generator (PEG) fabrication

PEGs were produced by sputter coating a metal electrode layer on both sides of the SEA extrusion printed $Ti_3C_2T_x$/PVDF-TrFE (0.00 wt% and 0.50 wt%) films, attaching wires onto each electrode, and subsequently encapsulating the entire device in insulating adhesive. The sputter coated (Nanochrome, Intlvac) electrodes consisted of a seeding chromium (Cr) layer and a gold (Au) layer, with a total thickness of 60 nm, deposited onto both sides through a shadow mask. The two electrode layers were deposited sequentially, without outgassing the chamber. The chamber pressure was maintained at 2 mTorr, with an argon (Ar) gas flow of 20 cm^3 min^{-1}. The Cr layer deposition utilized an AC dual magnetron source at 500 W power, with

a 10 s deposition time, corresponding to a Cr layer with thickness between 2 nm and 3 nm. The Au layer deposition utilized a DC magnetron source at 100 W power, with a 210 s deposition time, corresponding to an Au layer with thickness at 70 nm. The thickness of both layers was measured by AFM at 60 nm. The shadow mask was laser-cut from a 1 mm thick polymer sheet, pre-set for an opening with 1.5 cm length and 1.6 cm width, corresponding to an active area of 2.4 cm^2. Additional tabs were laser-cut adjacent to the width axis, with 0.5 cm length and 0.8 cm width, which were used for the attachment of wires and did not overlap between the two electrodes, therefore did not provide additional active area. The shadow mask design is shown in Supplementary Fig. 25a and the sputter coated SEA extrusion printed $Ti_3C_2T_x$/PVDF-TrFE (0.50 wt%) films are shown in Supplementary Fig. 25b.

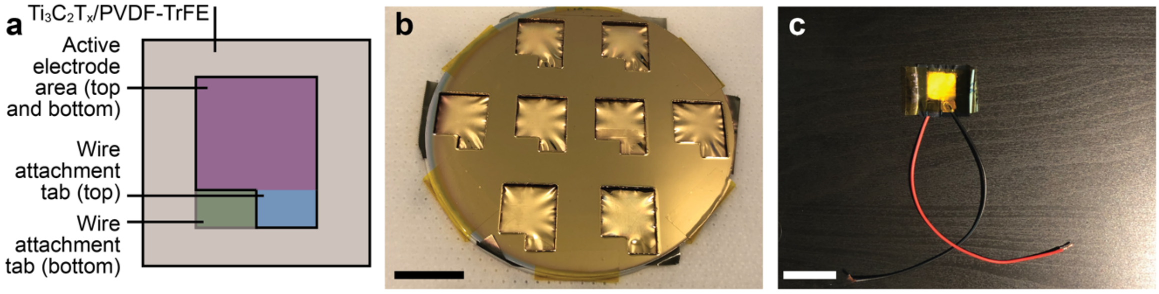

Supplementary Fig. 25: Piezoelectric generator layout. **a** Schematic showing the layout of the electrodes and the wire attachment tabs on both surfaces of the $Ti_3C_2T_x$/PVDF-TrFE films. **b** Photograph of the layout of the laser-cut shadow mask, with sputter-coated SEA extrusion printed $Ti_3C_2T_x$/PVDF-TrFE (0.50 wt%) films as the active layer of the PEGs. Scale bar represents 2 cm. **c** Photograph showing the final SEA extrusion printed $Ti_3C_2T_x$/PVDF-TrFE (0.50 wt%) PEG. Scale bar represents 3 cm.

The wires (FLEXI-E 0.15, Stäubli Electrical Connectors AG) were cut to a length of 15 cm, with 0.6 cm stripped and exposed at each end. One end of each wire was soldered to the non-adhesive side of copper (Cu) foil adhesive (1181, 3M), after cleaning the surface with propan-2-ol. Subsequently, the adhesive side of the Cu foil adhesive was attached to the designated tabs (Supplementary Fig. 25a) on each side of the sputter coated SEA extrusion printed $Ti_3C_2T_x$/PVDF-TrFE (0.00 wt% and 0.50 wt%) films. The final step in the PEG fabrication

was the encapsulation in insulating polyimide (Kapton) adhesive, which was adhered to both sides of the film to ensure no external electrical influences affect the macroscale energy harvesting experiments. A photograph of the completed PEG is shown in Supplementary Fig. 25c.

Macroscale displacement field measurement under compressive stress

The macroscale energy harvesting experiments were undertaken by the application of cyclic compression force and monitoring of the generated surface charge, configured to replicate the quasi-static Berlincourt method.[39] The cyclic compression was applied by a dynamic mechanical tester (ElectroForce 5500, TA Instruments) following a sinusoidal force pattern (Supplementary Fig. 29a, b). The surface charge was measured and converted to a voltage signal by a charge amplifier (Nexus 2692, Brüel & Kjær). The resultant voltage signal was logged directly to file by a data acquisition instrument (9223, National Instruments). The PVDF-TrFE PEGs possess a large source impedance,[10] between 1 MΩ and 10 MΩ, therefore the majority of voltage measurement techniques will introduce error arising from the capacitance of the connection cables and the input impedance of the measurement device. For these experiments, the charge amplifier was chosen as it eliminates these errors.[40,41]

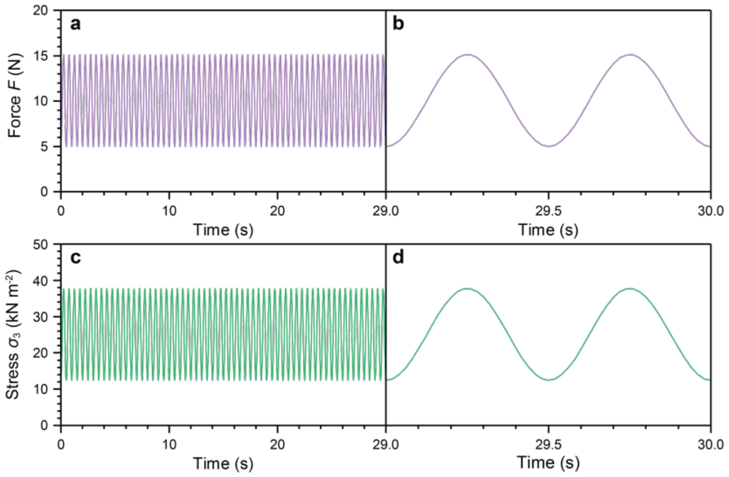

Supplementary Fig. 26: Representative mechanical excitation input. **a** The input force (F) over 30 s with frequency at 2 Hz and **b** the enlarged view of the final 1 s. **c** The input stress (σ_3), calculated from **a**, with **d** the enlarged view of the final 1 s.

The generated surface charge as a result of input force follows the constitutive equation for the direct piezoelectric effect,[16,37] given in Equation S9:

$$D_3 = d_{33}\sigma_3 + \varepsilon_{33}^{\sigma}E_3 \tag{S9}$$

Here, D_3 is the electric displacement field, d_{33} is the piezoelectric charge coefficient, σ_3 is the applied stress, $\varepsilon^{\sigma}{}_{33}$ is the dielectric permittivity at constant stress, E_3 is the electric field and the subscripts correspond to the directionality, in this instance all parallel to the thickness axis. Notably, in short circuit conditions where the input impedance of the load (in this instance the charge amplifier) is significantly lower than the output impedance of the PEG, the charge is transferred with no resistance.[41] In this instance, minimal voltage is generated and therefore $E_3 \approx 0$ V m^{-1}. Hence, the $\varepsilon^{\sigma}{}_{33}$ can be ignored and the expression takes on the form shown in Equation S10:

$$D_3 = d_{33}\sigma_3 \tag{S10}$$

The d_{33} can then be directly calculated from the input stress and the resultant electric displacement field, as shown in Equation S11:

$$d_{33} = \left(\frac{\partial D_3}{\partial \sigma_3}\right)^{E} \tag{S11}$$

Here, the superscript E denotes a constant electric field ($\partial E_3 = 0$). The stress is calculated from Equation S2 and is shown as a function of time in Supplementary Fig. 26c, d. The load cell of the mechanical tester, used to apply the stress, was cylindrical with a radius (r) of 12.5 mm. The compressive area (A_σ) (Supplementary Fig. 27, blue line, Supplementary Fig. 28, blue dashed line) was calculated individually for the samples, as the placement of the sputter coated electrodes was variable relative to the dimensions of the SEA extrusion printed films. The height (h) from the top of the Cu adhesive foil to the top of the sample (Supplementary Fig. 27) was measured (Supplementary Table 3) and the A_σ was calculated following Equation S12 for a circular segment:[42]

$$A_\sigma = \pi r^2 - \left(r \cos^{-1}\left(\frac{r-h}{r}\right) - (r-h)\sqrt{2rh - h^2} \right) \quad \text{(S12)}$$

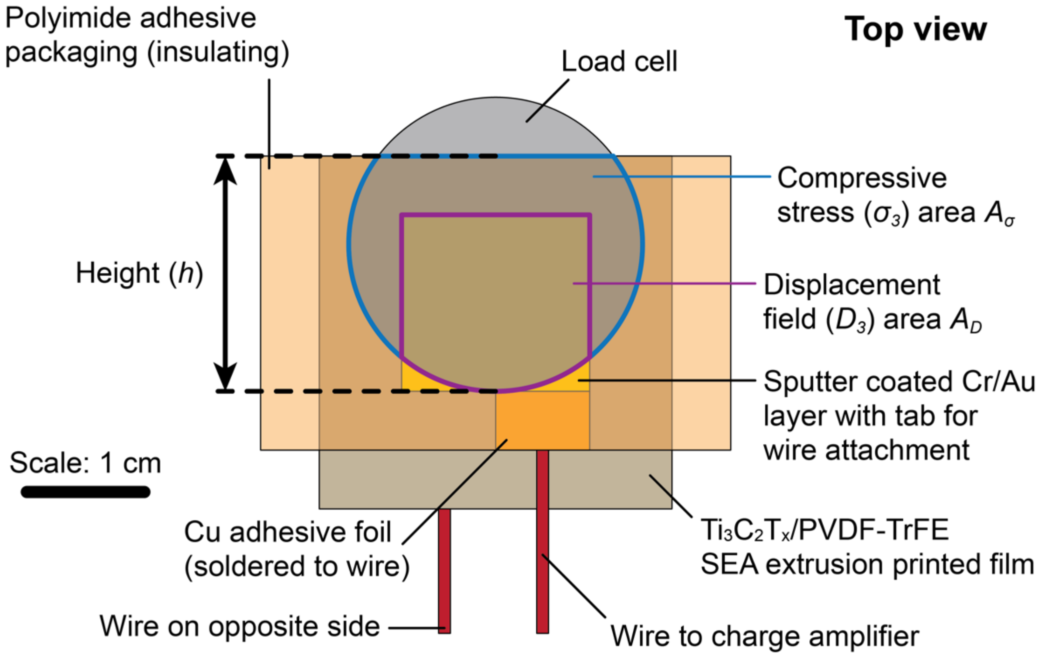

Supplementary Fig. 27: Schematic showing the layout of the $Ti_3C_2T_x$/PVDF-TrFE PEG for energy harvesting tests.

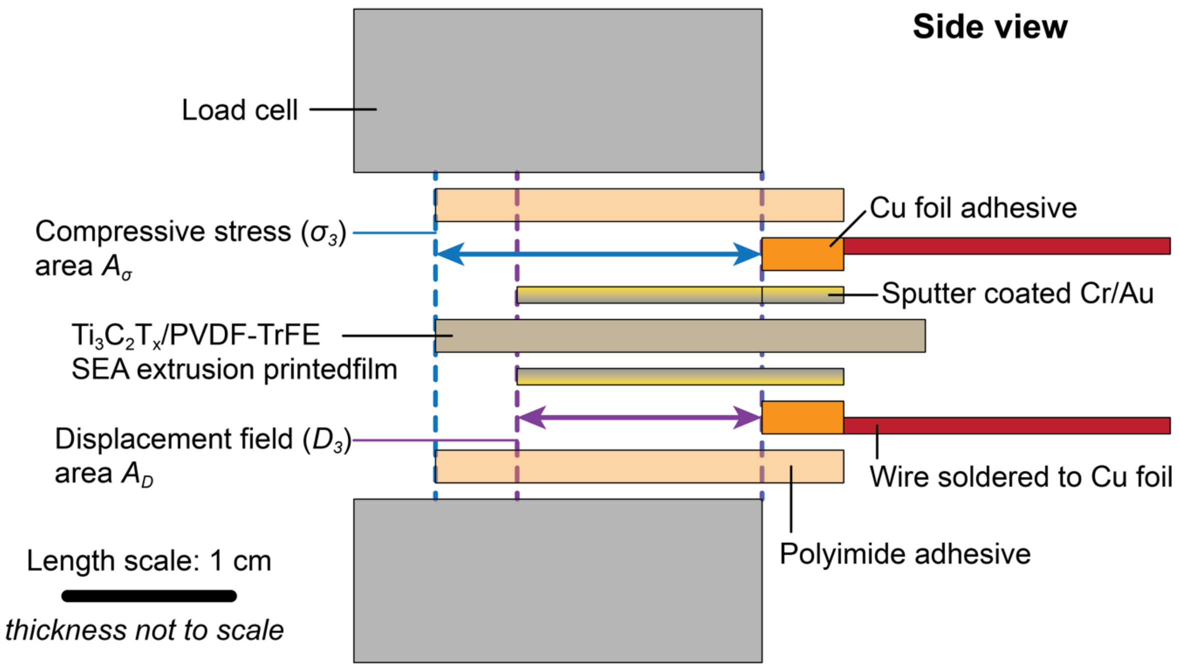

Supplementary Fig. 28: Schematic showing the side view of the components of the $Ti_3C_2T_x$/PVDF-TrFE PEG during energy harvesting characterization.

Supplementary Table 3: Values for the height (h) and the area under stress (A_σ) for energy harvesting experiments.

$Ti_3C_2T_x$ nanosheet concentration in PVDF-TrFE	h (mm)	A_σ (10^{-4} m^2)
0.00 wt%	6.0	4.00
0.50 wt%	5.3	4.15

Similarly, the electric displacement field is a value normalized to the active area (A_D), shown in Equation S12:

$$D_3 = \frac{Q}{A_D} \quad \text{(S12)}$$

In this instance, the A_D corresponded to the area with sputter coated electrodes on both sides, which is under impact (Supplementary Fig. 27, purple line, Supplementary Fig. 28, dashed purple line). The load cell was placed on the PEG such that the load cell did not make contact with the Cu foil adhesive (Supplementary Fig. 27, Supplementary Fig. 28). The A_D was measured as 2.25 cm^2 (2.25 x 10^{-4} m^2), based on the active electrode dimensions at 15 mm length and 16 mm width, whereby the major part of the electrode was compressed (Supplementary Fig. 27).

In order to ensure the generated charge arose only from the piezoelectric effect, the dependence of D_3 on σ_3 was investigated. In piezoelectric materials, as demonstrated in Equation S11, the slope must be linear, corresponding to the d_{33}. In the instances where the slope is not linear, either the constant E requirement is not satisfied, or contributions from contact electrification and/or flexoelectricity are present.[43–46] A representative SEA extrusion printed $Ti_3C_2T_x$/PVDF-TrFE (0.50 wt%) PEG was tested for this dependence. The minimum force was set at 5 N, to minimize the effects from contact electrification. The resultant data is shown in Supplementary Fig. 29. The slope was found to be linear, confirming the sole contribution of the direct piezoelectric effect to the measured surface charge.

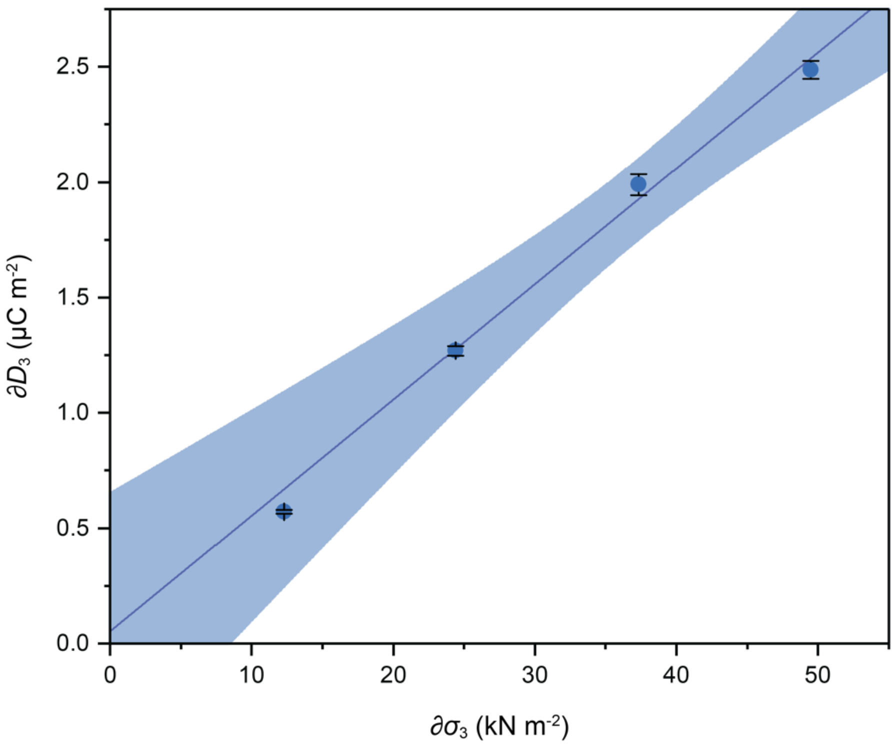

Supplementary Fig. 29: The generated electric displacement field (∂D_3) as a function of the input stress ($\partial \sigma_3$) for the SEA extrusion printed $Ti_3C_2T_x$/PVDF-TrFE (0.50 wt%) PEG. The solid line represents a linear fit to the data and the overlay represents a 95% confidence interval.

Upon the analysis of the generated surface charge (Q) in the SEA extrusion printed pristine PVDF-TrFE co-polymer PEG and the SEA extrusion printed $Ti_3C_2T_x$ /PVDF-TrFE (0.50 wt%) PEG (Fig. 5d), it was evident that a significant enhancement in the energy harvesting was observed upon the incorporation of the $Ti_3C_2T_x$ nanosheets into the PVDF-TrFE co-polymer. In this instance, the D_3 is related to the polarization (P_3) following equation S13:[37]

$$D_3 = \varepsilon_{33}^{\sigma} E_3 + P_3 \tag{S13}$$

Here, we were able to neglect the previously reported enhancements in the dielectric permittivity (ε) in $Ti_3C_2T_x$/fluoropolymer composites[44] due to the absence of external electric fields ($E \approx 0$ V m^{-1}), therefore the enhancements in Q (and subsequently D_3) were attributed directly to enhancements in the P_3. The polarization is given as the sum of the individual dipole moment vectors (μ_3) within a given volume (V), as shown in Equation S14, supporting the

enhanced dipole moment alignment within the materials, as the dipole moment magnitude and the volume were constant.

$$P_3 = \frac{\sum \mu_3}{V} \tag{S14}$$

The d_{33} of the $Ti_3C_2T_x$/PVDF-TrFE (0.50 wt%) PEG (at -52.0 pC N^{-1}) was found to be higher than that of completely poled PVDF-TrFE in literature (at approximately -38 pC N^{-1}),[16,48] suggesting that the electrical poling technique commonly utilized in literature does not completely polarize the pristine PVDF-TrFE co-polymer.[18,49] The presence of dielectric breakdown at a high poling electric field strength is hypothesized as the limiting factor in achieving completely polarized PVDF-TrFE for their utilization as PEGs.[50] Overcoming the limitation posed by the dielectric breakdown has profound opportunities in a multitude of fields where piezoelectric materials are used. The dipole locking mechanism from a nanomaterial template, as described in this study, has tremendous potential to unlock new applications for flexible piezoelectric materials, where the cost and energy input during manufacture is currently limiting commercial adoption.

Measurement of dielectric properties

The investigation of the dielectric properties of the SEA extrusion printed $Ti_3C_2T_x$/PVDF-TrFE was undertaken on the fabricated PEGs. An LCR meter (4284A, Hewlett Packard) was swept between 20 Hz and 1 MHz frequency at 0.5 V, with the probes connected directly to the wires of the PEG. Three individual PEG samples were measured at each $Ti_3C_2T_x$ nanosheet concentration. The measured capacitance (C) was normalized *via* Equation S15 to the thickness (t) and the overlapping electrode area (A_D) of the $Ti_3C_2T_x$/PVDF-TrFE film and the permittivity of free space ($\varepsilon_0 \approx 8.854 \times 10^{-12}$ F m^{-1}) in order to obtain the dielectric constant (ε_r):

$$\varepsilon_r = \frac{tC}{A\varepsilon_0} \tag{S15}$$

The resultant dependence of the ε_r on the frequency is shown in Supplementary Fig. 30a for the pristine PVDF-TrFE and the 0.50 wt% $Ti_3C_2T_x$/PVDF-TrFE films.

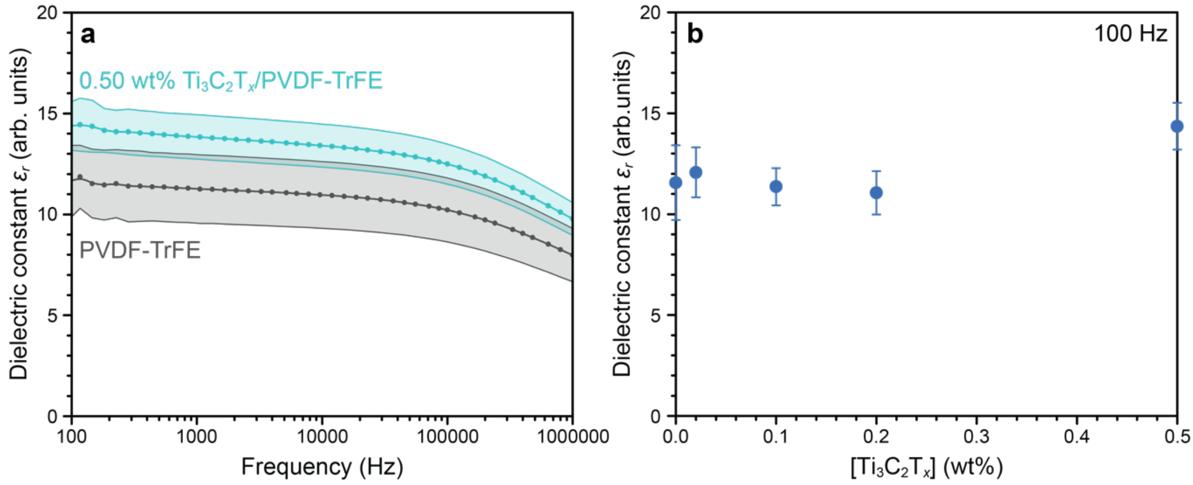

Supplementary Fig. 30: The dielectric constant for the SEA extrusion printed $Ti_3C_2T_x$/PVDF-TrFE composites, shown as a function of **a** frequency and **b** the $Ti_3C_2T_x$ concentration (100 Hz frequency). The error bars were obtained by testing three separate films at each $Ti_3C_2T_x$ concentration and represent the mean ± SD.

The ε_r was found to increase slightly between the pristine PVDF-TrFE film and the $Ti_3C_2T_x$/PVDF-TrFE film, exhibiting a similar response to an increasing frequency. Notably, the error between the samples was observed to overlap at all frequencies, therefore it was concluded that the increase was not significant. This is in accordance with the data recently presented by Tu *et al.*[47], demonstrating a 76% increase in the ε_r at 3.5 wt% of $Ti_3C_2T_x$ nanosheets (nanosheet size between 1 µm and 2 µm) in poly(vinylidene fluoride-trifluoroethylene-chlorofluoroethylene) (PVDF-TrFE-CFE), a ter-polymer of the PVDF-TrFE co-polymer. Notably, the nanosheet dimensions in this study are significantly smaller in size (approximately 300 nm) and the maximum $Ti_3C_2T_x$ nanosheet concentration is significantly lower (0.5 wt%), thus the lower increase in the ε_r of the composites presented in this manuscript is expected. Furthermore, the trend of the ε_r with increasing $Ti_3C_2T_x$ nanosheet concentration (Supplementary Fig. 30b) exhibits little correlation between the two parameters, and the ε_r of the 0.20 wt% $Ti_3C_2T_x$/PVDF-TrFE film is equal to that of the pristine PVDF-TrFE film. This

data confirms that the increase in the d_{33} of the $Ti_3C_2T_x$/PVDF-TrFE composites does not arise from an increased ε_r, as discussion in the previous section.

Piezoelectric voltage coefficient and piezoelectric figure of merit

The measurement of the d_{33} by the macroscale (Berlincourt) method and the determination of the ε_r enables the subsequent calculation of the piezoelectric voltage coefficient (g_{33}) and consequently the piezoelectric figure of merit (FOM).[16,39] The g_{33} is calculated following Equation S16, corresponding to the generated Q data presented in Fig. 5d.

$$g_{33} = \frac{d_{33}}{\varepsilon_{33}^{\sigma}} = \frac{d_{33}}{\varepsilon_r \varepsilon_0} \tag{S16}$$

The average g_{33} of the SEA extrusion printed pristine PVDF-TrFE PEG, measured from 60 individual compression cycles, was found to be 341 mV m N^{-1}. As expected, the partial polarization from the shear stress at the nozzle wall during the SEA extrusion printing process results in a g_{33} value lower than that of literature reports for electrically poled PVDF-TrFE (approximately 380 mV m N^{-1}).[16] More importantly, the SEA extrusion printed 0.50 wt% $Ti_3C_2T_x$/PVDF-TrFE PEG exhibited a g_{33} value of 402 mV m N^{-1}, larger in comparison to both the pristine PVDF-TrFE film prepared in this manuscript and the electrically poled PVDF-TrFE found in literature.

Moreover, the FOM for the SEA extrusion printed $Ti_3C_2T_x$/PVDF-TrFE was subsequently calculated using Equation S17:

$$\text{FOM} = d_{33} g_{33} = \frac{d_{33}^2}{\varepsilon_{33}^{\sigma}} \tag{S17}$$

The FOM was calculated for each of the 60 mechanical compression cycles corresponding to the Q measurements in Fig. 5d, shown in Supplementary Fig. 31 for the SEA extrusion printed

PEGs containing 0.00 wt% and 0.50 wt% $Ti_3C_2T_x$ nanosheets, as well as the literature value for poled PVDF-TrFE films.

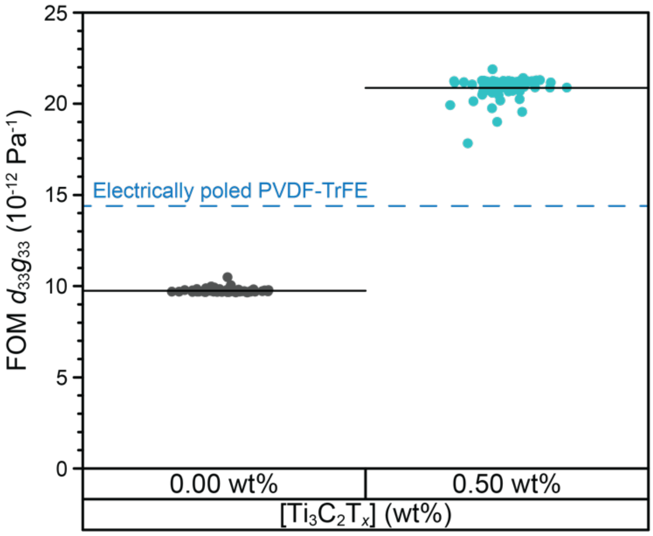

Supplementary Fig. 31: The piezoelectric figure of merit (FOM) of the SEA extrusion printed $Ti_3C_2T_x$/PVDF-TrFE PEGs, demonstrated for the pristine PVDF-TrFE (0.00 wt% $Ti_3C_2T_x$, gray circles) and the 0.50 wt% $Ti_3C_2T_x$ (teal circles) PEGs. Each data point corresponds to one mechanical compression cycle for the data presented in Fig. 5d. The horizontal bars represent the average for 60 compression cycles. The dashed blue line corresponds to the literature value for the FOM of electrically poled PVDF-TrFE.

The average FOM for the SEA extrusion printed pristine PVDF-TrFE PEG was calculated as 9.7 x 10^{-12} Pa^{-1}. As expected, the lower d_{33} and g_{33} of the pristine PVDF-TrFE PEG relative to literature values for electrically poled PVDF-TrFE (14.4 x 10^{-12} Pa^{-1}) results in a 33% lower FOM. Conversely, the FOM of the 0.50 wt% $Ti_3C_2T_x$/PVDF-TrFE PEG at 20.9 x 10^{-12} Pa^{-1} is significantly higher (45%) than electrically poled PVDF-TrFE, which is largely attributed to the strong electrostatic interactions between the PVDF-TrFE co-polymer and the $Ti_3C_2T_x$ nanosheets. Further, the FOM of the 0.50 wt% $Ti_3C_2T_x$/PVDF-TrFE PEG is 115% higher than the SEA extrusion printed pristine PVDF-TrFE PEG. Unlike the pristine PVDF-TrFE PEG, which is restricted to the SEA extrusion printing process in order to exhibit partial polarization from induced shear stresses during deposition, the polarization-locked $Ti_3C_2T_x$/PVDF-TrFE

solution possesses the flexibility for processing *via* conventional polymer film deposition techniques (e.g., solvent casting) while retaining the high piezoelectric properties (as demonstrated by PFM in Fig. 5c). Importantly, the FOM of the $Ti_3C_2T_x$/PVDF-TrFE PEG is 45% higher than electrically poled PVDF-TrFE, demonstrating a viable low energy deposition technique to produce effective flexible piezoelectric energy harvesting devices on a mass-produced scale.

The comparison of the polarization-locked $Ti_3C_2T_x$/PVDF-TrFE PEGs presented in this work to piezoelectric materials reported in literature (Supplementary Table 4) demonstrates that the polarization-locked PEG possesses the highest g_{33} and FOM values reported to date, taking into account the analysis of Deutz *et al.*[39] and van den Ende *et al.*[51], which investigate perovskite structure materials and polymer-perovskite composites, respectively.

Table S4: Comparison of the piezoelectric charge coefficient (d_{33}), piezoelectric voltage coefficient (g_{33}) and the piezoelectric figure of merit (FOM) between the SEA extrusion printed films presented in the manuscript and fluoropolymer-based materials found in literature for which the d_{33} and g_{33} was presented.

Material	Processing	Poling	$\lvert d_{33}\rvert$ (pC N^{-1})	g_{33}† (mV m N^{-1})	FOM‡ (x 10^{-12} Pa^{-1})	Ref.
**PVDF-TrFE**	**Extrusion printing**	**None**	**29**	**341**	**9.7**	**This work**
**$Ti_3C_2T_x$/PVDF-TrFE (0.50 wt%)**	**Extrusion printing**	**None**	**52**	**402**	**20.9**	**This work**
ZnO/PVDF*	Drop casting	None	50	219	11.0	[52]
MnO_2/PVDF	Electrospinning, hot pressing and rolling	80 MV m^{-1}, 80 °C, 2 h	38	318	12.1	[53]
BCZT50/PVDF*	Melt mixing	20 MV m^{-1}, 70 °C, 0.5 h	27	114	3.1	[54]
PVDF-TrFE	Spin coating	200 MV m^{-1}	22	187	4.0	[55]
PVDF	Solvent casting + double rolling	170 MV m^{-1}, 70 °C, 1.2 h	29	234	6.8	[56]
MWCNT/PVDF	Solvent casting + double rolling	100 MV m^{-1}, 70 °C, 1.5 h	33	169	5.6	[57]
PVDF-HFP	Blade coating	0.5 MV m^{-1}, 100 °C, 0.5 h	11	113	1.2	[58]
PVDF-TrFE	Solvent casting	105 MV m^{-1}, 100 °C	23	236	5.4	[59]
BT/PVDF-TrFE*	Solvent casting	10 MV m^{-1}, 110 °C, 0.5 h	34	37	1.3	[60]

† Calculated *via* Equation S16;
‡ Calculated *via* Equation S17;
* Additive has been reported to exhibit piezoelectric properties;

References

1. Zhang, J. *et al.* Highly Conductive $Ti_3C_2T_x$ MXene Hybrid Fibers for Flexible and Elastic Fiber-Shaped Supercapacitors. *Small* **15**, 1804732 (2019).
2. Lukatskaya, M. R. *et al.* Ultra-high-rate pseudocapacitive energy storage in two-dimensional transition metal carbides. *Nat. Energy* **6**, (2017).
3. Xia, Y. *et al.* Thickness-independent capacitance of vertically aligned liquid-crystalline MXenes. *Nature* **557**, 409–412 (2018).
4. Lukatskaya, M. R. *et al.* Cation intercalation and high volumetric capacitance of two-dimensional titanium carbide. *Science* **341**, 1502–1505 (2013).
5. Zhang, J. *et al.* Scalable Manufacturing of Free-Standing, Strong $Ti_3C_2T_x$ MXene Films with Outstanding Conductivity. *Adv. Mater.* **32**, 2001093 (2020).
6. Zhang, C. J. *et al.* Oxidation Stability of Colloidal Two-Dimensional Titanium Carbides (MXenes). *Chem. Mater.* **29**, 4848–4856 (2017).
7. Rosenkranz, A. *et al.* Multi-layer $Ti_3C_2T_x$-nanoparticles (MXenes) as solid lubricants – Role of surface terminations and intercalated water. *Appl. Surf. Sci.* **494**, 13–21 (2019).
8. Halim, J. *et al.* X-ray photoelectron spectroscopy of select multi-layered transition metal carbides (MXenes). *Appl. Surf. Sci.* **362**, 406–417 (2016).
9. Sherrell, P. C. *et al.* Thickness-Dependent Characterization of Chemically Exfoliated TiS_2 Nanosheets. *ACS Omega* **3**, 8655–8662 (2018).
10. Shepelin, N. A. *et al.* Printed recyclable and self-poled polymer piezoelectric generators through single-walled carbon nanotube templating. *Energy Environ. Sci.* **13**, 868–883 (2020).
11. Yamada, N., Shiratori, A. & Murasawa, G. A poly(vinylidene fluoride) film printing system using solution droplet casting. *Smart Mater. Struct.* **28**, 044003 (2019).
12. Bipp, H. & Kieczka, H. Formamides. in *Ullman's Encyclopedia of Industrial Chemistry*

(Wiley-VCH, Weinheim, 2011).

13. Smallwood, I. M. *Handbook of organic solvent properties* (John Wiley & Sons, New York, 1996).

14. Sifniades, S. & Levy, A. B. Acetone. in *Ullmann's Encyclopedia of Industrial Chemistry* (Wiley-VCH, Weinheim, 2000).

15. Shepelin, N. A. *et al.* 3D printing of poly(vinylidene fluoride-trifluoroethylene): a poling-free technique to manufacture flexible and transparent piezoelectric generators. *MRS Commun.* **9**, 159–164 (2019).

16. Shepelin, N. A. *et al.* New developments in composites, copolymer technologies and processing techniques for flexible fluoropolymer piezoelectric generators for efficient energy harvesting. *Energy Environ. Sci.* **12**, 1143–1176 (2019).

17. Martins, P., Lopes, A. C. C. & Lanceros-Mendez, S. Electroactive phases of poly(vinylidene fluoride): Determination, processing and applications. *Prog. Polym. Sci.* **39**, 683–706 (2014).

18. Lovinger, A. J. Ferroelectric polymers. *Science* **220**, 1115–1121 (1983).

19. Li, L., Lin, Q., Tang, M., Duncan, A. J. E. & Ke, C. Advanced Polymer Designs for Direct-Ink-Write 3D Printing. *Chem. Eur. J.* **25**, 10768–10781 (2019).

20. Gratson, G. M. & Lewis, J. A. Phase behavior and rheological properties of polyelectrolyte inks for direct-write assembly. *Langmuir* **21**, 457–464 (2005).

21. Bottino, A., Capannelli, G., Munari, S. & Turturro, A. Solubility parameters of poly(vinylidene fluoride). *J. Polym. Sci. B* **26**, 785–794 (1988).

22. Wagner, M. H. & Meissner, J. Network disentanglement and time-dependent flow behaviour of polymer melts. *Makromol. Chem.* **181**, 1533–1550 (1980).

23. Kavanagh, G. M. & Ross-Murphy, S. B. Rheological characterisation of polymer gels. *Prog. Polym. Sci.* **23**, 533–562 (1998).

24. Gao, P. & Mackley, M. R. General model for the diffusion and swelling of polymers and its application to ultra-high molecular mass polyethylene. *Proc. R. Soc. A* **444**, 267–285 (1994).

25. Kjøniksen, A. L., Nyström, B. & Lindman, B. Dynamic viscoelasticity of gelling and nongelling aqueous mixtures of ethyl(hydroxyethyl)cellulose and an ionic surfactant. *Macromolecules* **31**, 1852–1858 (1998).

26. American Society for Testing and Materials. *ASTM D1003-13, Standard Test Method for Haze and Luminous Transmittance of Transparent Plastics*. (2013).

27. Sarycheva, A. & Gogotsi, Y. Raman Spectroscopy Analysis of the Structure and Surface Chemistry of $Ti_3C_2T_x$ MXene. *Chem. Mater.* **32**, 3480–3488 (2020).

28. Weerasinghe, A., Lu, C.-T., Maroudas, D. & Ramasubramaniam, A. Multiscale Shear-Lag Analysis of Stiffness Enhancement in Polymer-Graphene Nanocomposites. *ACS Appl. Mater. Interfaces* **9**, 23092–23098 (2017).

29. Beck, M. E. & Hersam, M. C. Emerging Opportunities for Electrostatic Control in Atomically Thin Devices. *ACS Nano* **14**, 6498-6518 (2020).

30. Su, R. *et al.* Ferroelectric behavior in the high temperature paraelectric phase in a poly(vinylidene fluoride-co-trifluoroethylene) random copolymer. *Polymer* **53**, 728–739 (2012).

31. García-Gutiérrez, M.-C. *et al.* Understanding crystallization features of P(VDF-TrFE) copolymers under confinement to optimize ferroelectricity in nanostructures. *Nanoscale* **5**, 6006–6012 (2013).

32. Bellet-Amalric, E. & Legrand, J. F. Crystalline structures and phase transition of the ferroelectric P(VDF-TrFE) copolymers, a neutron diffraction study. *Eur. Phys. J. B* **3**, 225–236 (1998).

33. Piedrahita-Bello, M. *et al.* Mechano-electric coupling in P(VDF–TrFE)/spin crossover

composites. *J. Mater. Chem. C* **8**, 6042–6051 (2020).

34. Cebe, P. & Runt, J. P(VDF-TrFE)-layered silicate nanocomposites. Part 1. X-ray scattering and thermal analysis studies. *Polymer* **45**, 1923–1932 (2004).

35. Chen, X. *et al.* Self-powered flexible pressure sensors with vertically well-aligned piezoelectric nanowire arrays for monitoring vital signs. *J. Mater. Chem. C* **3**, 11806–11814 (2015).

36. Kholkin, A. L., Kalinin, S. V., Roelofs, A. & Gruverman, A. Review of Ferroelectric Domain Imaging by Piezoresponse Force Microscopy. in *Scanning Probe Microscopy* (eds. Kalinin, S. & Gruverman, A.) 173–214 (Springer, 2007).

37. *ANSI/IEEE 176-1987 Standard on Piezoelectricity*. (1988). doi:10.1109/IEEESTD.1988.79638

38. Gruverman, A., Alexe, M. & Meier, D. Piezoresponse force microscopy and nanoferroic phenomena. *Nat. Commun.* **10**, 1661 (2019).

39. Deutz, D. B. *et al.* Analysis and experimental validation of the figure of merit for piezoelectric energy harvesters. *Mater. Horizons* **5**, 444–453 (2018).

40. Fleming, A. J. & Moheimani, S. O. R. Precision current and charge amplifiers for driving highly capacitive piezoelectric loads. *Electron. Lett.* **39**, 282–284 (2003).

41. Lee, B. Y. *et al.* Virus-based piezoelectric energy generation. *Nat. Nanotechnol.* **7**, 351–356 (2012).

42. Harris, J. W. & Stöcker, H. Segment of a Circle. in *Handbook of Mathematics and Computational Science* 92–93 (Springer-Verlag, 1998).

43. Šutka, A. *et al.* Measuring Piezoelectric Output—Fact or Friction? *Adv. Mater.* **32**, 2002979 (2020).

44. Zhou, L., Liu, D., Wang, J. & Wang, Z. L. Triboelectric nanogenerators: Fundamental physics and potential applications. *Friction* **8**, 481–506 (2020).

45. Ahmed, A. *et al.* Integrated Triboelectric Nanogenerators in the Era of the Internet of Things. *Adv. Sci.* **6**, 1802230 (2019).

46. Nguyen, T. D., Mao, S., Yeh, Y. W., Purohit, P. K. & McAlpine, M. C. Nanoscale flexoelectricity. *Adv. Mater.* **25**, 946–974 (2013).

47. Tu, S. *et al.* Enhancement of Dielectric Permittivity of $Ti_3C_2T_x$ MXene/Polymer Composites by Controlling Flake Size and Surface Termination. *ACS Appl. Mater. Interfaces* **11**, 27358–27362 (2019).

48. Bowen, C. R., Kim, H. A., Weaver, P. M. & Dunn, S. Piezoelectric and ferroelectric materials and structures for energy harvesting applications. *Energy Environ. Sci.* **7**, 25–44 (2014).

49. Liu, Y. *et al.* Ferroelectric polymers exhibiting behaviour reminiscent of a morphotropic phase boundary. *Nature* **562**, 96–100 (2018).

50. Claude, J., Lu, Y., Li, K. & Wang, Q. Electrical storage in poly(vinylidene fluoride) based ferroelectric polymers: Correlating polymer structure to electrical breakdown strength. *Chem. Mater.* **20**, 2078–2080 (2008).

51. Stuber, V. L. *et al.* Flexible Lead-Free Piezoelectric Composite Materials for Energy Harvesting Applications. *Energy Technol.* **7,** 177-185 (2019).

52. Thakur, P. *et al.* Superior performances of *in situ* synthesized ZnO/PVDF thin film based self-poled piezoelectric nanogenerators and self-charged photo-power bank with high durability. *Nano Energy* **44,** 456-467 (2018).

53. Zhao, Q. *et al.* Flexible textured MnO_2 nanorods/PVDF hybrid films with superior piezoelectric performance for energy harvesting application. *Compos. Sci. Technol.* **199,** 108330 (2020).

54. Kumar, A., Kumar, A. & Prasad, K. Power generation characteristics of $0.50(Ba_{0.7}Ca_{0.3})TiO_3$-$0.50Ba(Zr_{0.2}Ti_{0.8})O_3$/PVDF nanocomposites under impact

loading. *J. Mater. Sci. Mater. Electron.* **31,** 12708-12714 (2020).

55. Zhou, Z. *et al.* Enhanced piezoelectric and acoustic performances of poly(cinylidene fluoride-trifluoroethylene) films for hydroacoustic applications. *Phys. Chem. Chem. Phys.* **22,** 5711-5722 (2020).

56. Yang, L. *et al.* Effect of rolling temperature on the microstructure and electric properties of β-polyinylidene fluoride films. *J. Mater. Sci. Mater. Electron.* **29,** 15957-15965 (2018).

57. Yang, L., *et al.* Enhanced electrical properties of multiwalled carbon nanotube/poly(vinylidenefluoride) films through a rolling process. *J. Mater. Sci. Mater. Electron.* **25,** 2126-2137 (2014).

58. Sousa, R. E., *et al.* Microstructural variations of poly(vinylidene fluoride co-hexafluoropropylene) and their influence on the thermal, dielectric and piezoelectric properties. *Polym. Test.* **40,** 245-255 (2014).

59. Xia, W., *et al.* Dielectric, piezoelectric and ferroelectric properties of a poly(vinylidene fluoride-*co*-trifluoroethylene) synthesized via a hydrogenation process. *Polymer* **54,** 440-446 (2013).

60. Vacche, S. D., *et al.* The effect of processing conditions on the morphology, thermomechanical, dielectric and piezoelectric properties of P(VDF-TrFE)/$BaTiO_3$ composites. *J. Mater. Sci.* **47,** 4763-4774 (2012).
