# Supplementary material for: Interfacial piezoelectric polarization locking in printable Ti3C2Tx MXene-fluoropolymer composites
Source: Nat Commun. 2021 May 26;12:3171. doi: 10.1038/s41467-021-23341-3 (PMC8155213; doi:10.1038/s41467-021-23341-3)
Supplement: Supplementary file 5 — Description of Additional Supplementary Files [file 41467_2021_23341_MOESM5_ESM.docx]

Description of additional supplementary information files

Title: Supplementary Movie 1

Desscription: Temporal evolution of the PVDF-TrFE co-polymer polarization vector direction adsorbed on a Ti3C2Tx nanosheet substrate (left) and graphene substrate (right) over a 1.6 ns timespan. The PVDF-TrFE film consists of 70 co-polymer chains.

Title: Supplementary Movie 2

Description: Temporal evolution of the individual-chain polarization vector direction in the PVDF-TrFE co-polymer (5 chains shown from a total of 70 chains), adsorbed on a Ti3C2Tx nanosheet substrate (left) and graphene substrate (right) over a 1.6 ns timespan.
